# Effectiveness of Fosfomycin for the Treatment of Multidrug-Resistant *Escherichia coli* Bacteremic Urinary Tract Infections: A Randomized Clinical Trial

**DOI:** 10.1001/jamanetworkopen.2021.37277

**Published:** 2022-01-13

**Authors:** Jesús Sojo-Dorado, Inmaculada López-Hernández, Clara Rosso-Fernandez, Isabel M. Morales, Zaira R. Palacios-Baena, Alicia Hernández-Torres, Esperanza Merino de Lucas, Laura Escolà-Vergé, Elena Bereciartua, Elisa García-Vázquez, Vicente Pintado, Lucía Boix-Palop, Clara Natera-Kindelán, Luisa Sorlí, Nuria Borrell, Livia Giner-Oncina, Concha Amador-Prous, Evelyn Shaw, Alfredo Jover-Saenz, Jose Molina, Rosa M. Martínez-Alvarez, Carlos J. Dueñas, Jorge Calvo-Montes, Jose T. Silva, Miguel A. Cárdenes, María Lecuona, Virginia Pomar, Lucía Valiente de Santis, Genoveva Yagüe-Guirao, María Angeles Lobo-Acosta, Vicente Merino-Bohórquez, Alvaro Pascual, Jesús Rodríguez-Baño, and the REIPI-GEIRAS-FOREST group

**Affiliations:** 1 Unidad Clínica de Enfermedades Infecciosas y Microbiología, Hospital Universitario Virgen Macarena, Instituto de Biomedicina de Sevilla, Consejo Superior de Investigaciones Científicas, Departamentos de Medicina y Microbiología, Universidad de Sevilla, Sevilla, Spain; 2 Unidad de Investigación Clínica y Ensayos Clínicos, Hospital Universitario Virgen del Rocío-Macarena, Sevilla, Spain; 3 Unidad Clínica de Urgencias, Hospital Universitario Virgen Macarena, Sevilla, Spain; 4 Servicio de Medicina Interna, Unidad de Enfermedades Infecciosas, Hospital Clínico Universitario Virgen de la Arrixaca, Murcia, Spain; 5 Unidad de Enfermedades Infecciosas, Hospital General Universitario de Alicante, Instituto Investigación Biomédica de Alicante, Alicante, Spain; 6 Servicio de Enfermedades Infecciosas, Hospital Universitario Vall d’Hebrón, Barcelona, Spain; 7 Unidad de Enfermedades Infecciosas, Hospital Universitario Cruces, Instituto de Investigación Biocruces, Baracaldo, Vizcaya, Spain; 8 Servicio de Enfermedades Infecciosas, Hospital Universitario Ramón y Cajal and Instituto Ramón y Cajal de Investigación Sanitaria, Madrid, Spain; 9 Unidad de Enfermedades Infecciosas, Hospital Universitari Mútua Terrassa, Terrassa, Barcelona, Spain; 10 Unidad de Gestión Clínica de Enfermedades Infecciosas, Hospital Universitario Reina Sofía, Córdoba, Spain; 11 Servicio de Enfermedades Infecciosas, Hospital del Mar, and Grupo de Investigación en Patología Infecciosa y Antibioterapia, Institut Hospital del Mar d'Investigacions Mèdiques, Universitat Pompeu Fabra, Barcelona, Spain; 12 Servicio de Microbiología, Hospital Universitario Son Espases, Palma de Mallorca, Spain; 13 Unidad de Enfermedades Infecciosas, Hospital Marina Baixa, Villajoyosa, Alicante, Spain; 14 Servei de Malalties Infeccioses, Hospital Universitari de Bellvitge, Epidemiologia de les Infeccions Bacterianes, Patologia Infecciosa i Transplantament, Institut d’Investigació Biomèdica de Bellvitge (IDIBELL), L’Hospitalet de Llobregat, Barcelona, Spain; 15 Unidad Territorial Infección Nosocomial, Hospital Universitari Arnau de Vilanova, Institut de Recerca Biomèdica de Lleida, Lleida, Spain; 16 Unidad Clínica de Enfermedades Infecciosas, Microbiología y Medicina Preventiva, Hospital Universitario Virgen del Rocío, Instituto de Biomedicina de Sevilla, Consejo Superior de Investigaciones Científicas, Departamentos de Medicina y Microbiología, Universidad de Sevilla, Sevilla, Spain; 17 Unidad de Enfermedades Infecciosas, Hospital Royo Villanova, Zaragoza, Spain; 18 Now with Unidad de Enfermedades Infecciosas, Hospital Miguel Servet, Zaragoza, Spain; 19 Unidad de Enfermedades Infecciosas, Hospital Universitario de Burgos, Burgos, Spain; 20 Presently with Unidad de Enfermedades Infecciosas, Hospital Clínico Universitario de Valladolid, Valladolid, Spain; 21 Servicio de Microbiología, Hospital Universitario Marqués de Valdecilla, Instituto de Investigación Sanitaria Valdecilla, Santander, Spain; 22 Unidad de Enfermedades Infecciosas, Hospital Universitario Doce de Octubre, Madrid, Spain; 23 Unidad de Enfermedades Infecciosas, Hospital Universitario de Gran Canaria Dr Negrín, Las Palmas de Gran Canaria, Spain; 24 Servicio de Microbiología y Control de la Infección, Hospital Universitario de Canarias, La Laguna, Spain; 25 Unidad de Enfermedades Infecciosas, Servicio de Medicina Interna, Hospital de la Santa Creu i Sant Pau, Barcelona, Spain; 26 Servicio de Enfermedades Infecciosas, UGC de Enfermedades Infecciosas, Microbiología y Medicina Preventiva, Hospital Regional Universitario de Málaga, Instituto de Investigación Biomédica de Málaga, Málaga, Spain; 27 Servicio de Microbiología, Hospital Clínico Universitario Virgen de la Arrixaca, Instituto Murciano de Investigación Biosanitaria, Murcia, Spain; 28 Unidad Clínica de Farmacia, Hospital Universitario Virgen Macarena and Departamento de Farmacología, Universidad de Sevilla, Sevilla, Spain

## Abstract

**Importance:**

The consumption of broad-spectrum drugs has increased as a consequence of the spread of multidrug-resistant (MDR) *Escherichia coli*. Finding alternatives for these infections is critical, for which some neglected drugs may be an option.

**Objective:**

To determine whether fosfomycin is noninferior to ceftriaxone or meropenem in the targeted treatment of bacteremic urinary tract infections (bUTIs) due to MDR *E coli*.

**Design, Setting, and Participants:**

This multicenter, randomized, pragmatic, open clinical trial was conducted at 22 Spanish hospitals from June 2014 to December 2018. Eligible participants were adult patients with bacteremic urinary tract infections due to MDR *E coli*; 161 of 1578 screened patients were randomized and followed up for 60 days. Data were analyzed in May 2021.

**Interventions:**

Patients were randomized 1 to 1 to receive intravenous fosfomycin disodium at 4 g every 6 hours (70 participants) or a comparator (ceftriaxone or meropenem if resistant; 73 participants) with the option to switch to oral fosfomycin trometamol for the fosfomycin group or an active oral drug or parenteral ertapenem for the comparator group after 4 days.

**Main Outcomes and Measures:**

The primary outcome was clinical and microbiological cure (CMC) 5 to 7 days after finalization of treatment; a noninferiority margin of 7% was considered.

**Results:**

Among 143 patients in the modified intention-to-treat population (median [IQR] age, 72 [62-81] years; 73 [51.0%] women), 48 of 70 patients (68.6%) treated with fosfomycin and 57 of 73 patients (78.1%) treated with comparators reached CMC (risk difference, −9.4 percentage points; 1-sided 95% CI, −21.5 to ∞ percentage points; *P* = .10). While clinical or microbiological failure occurred among 10 patients (14.3%) treated with fosfomycin and 14 patients (19.7%) treated with comparators (risk difference, −5.4 percentage points; 1-sided 95% CI, −∞ to 4.9; percentage points; *P* = .19), an increased rate of adverse event–related discontinuations occurred with fosfomycin vs comparators (6 discontinuations [8.5%] vs 0 discontinuations; *P* = .006). In an exploratory analysis among a subset of 38 patients who underwent rectal colonization studies, patients treated with fosfomycin acquired a new ceftriaxone-resistant or meropenem-resistant gram-negative bacteria at a decreased rate compared with patients treated with comparators (0 of 21 patients vs 4 of 17 patients [23.5%]; 1-sided *P* = .01).

**Conclusions and Relevance:**

This study found that fosfomycin did not demonstrate noninferiority to comparators as targeted treatment of bUTI from MDR *E coli*; this was due to an increased rate of adverse event–related discontinuations. This finding suggests that fosfomycin may be considered for selected patients with these infections.

**Trial Registration:**

ClinicalTrials.gov Identifier: NCT02142751

## Key Points

**Question:**

Is fosfomycin noninferior to ceftriaxone or meropenem for bacteremic urinary tract infections due to multidrug-resistant *Escherichia coli*?

**Findings:**

In this randomized clinical trial including 143 adults with multidrug-resistant bacteremic urinary tract infections due to *E coli*, clinical and microbiological cure was achieved by 68.6% of patients treated with fosfomycin and 78.1% of patients treated with comparators, with fosfomycin not reaching noninferiority. This was due to an increased rate of adverse event–related discontinuations with fosfomycin (8.5% vs 0%).

**Meaning:**

While fosfomycin did not demonstrate noninferiority, the findings of this study suggest that it may still be considered among selected patients.

## Teaser

This randomized clinical trial investigates noninferiority of fosfomycin vs ceftriaxone or meropenem in achieving clinical and microbiological cure among patients with urinary tract infections due to multidrug-resistant *Escherichia coli*.

## Introduction

*Escherichia coli* is one of the most frequently occurring human pathogens. After a massive use of cephalosporins and fluoroquinolones, multidrug-resistant (MDR) isolates have spread dramatically worldwide.^1,2^ As a consequence, the consumption of last-resort drugs, such as carbapenems, increased over the last 20 years,^3^ which in turn is facilitating the dramatic spread of carbapenem-resistance.^4^ These outcomes suggest that finding alternatives for the treatment of MDR *E coli* infections is a medical need.

Some old drugs were inadequately developed according to present standards, which suggests that appropriate trials must be performed to evaluate the potential efficacy of these drugs. Targeted therapy is a potential indication for these drugs, allowing a decreased consumption of broad-spectrum drugs. Fosfomycin, discovered more than 40 years ago, is active against a wide range of pathogens, including MDR Enterobacterales.^5,6^ This drug is available for intravenous use as fosfomycin disodium in some countries (although not in the United States) and as an oral formulation (ie, fosfomycin trometamol). However, high-quality studies with fosfomycin are scarce.^5,7^ Recently, it was shown to be noninferior to piperacillin-tazobactam for treatment of complicated urinary tract infections (cUTI).^8^ Because cUTI includes highly heterogeneous infection types and considering that fosfomycin may be less efficacious against Enterobacterales than against other than *E coli*,^9,10^ we conducted this study to test the hypothesis that fosfomycin is not inferior to ceftriaxone or meropenem for the targeted treatment of bacteremic UTI (bUTI) caused by MDR *E coli*.

## Methods

The Fosfomycin vs Meropenem or Ceftriaxone in Bacteriemic Infections Caused by Multidrug Resistance in *E. Coli* (FOREST) randomized clinical trial was conceived as a noninferiority trial intended to provide information on fosfomycin as an alternative drug to ceftriaxone and carbapenems, which are associated with increased risk of colonization and infection due to MDR bacteria; therefore, treatment with fosfomycin may have a protective effect for that risk. The Andalusian Ethics Committee approved this study, and written informed consent was obtained from all participants. The results are reported according to the Consolidated Standards of Reporting Trials (CONSORT) reporting guideline.

### Study Design and Patients

FOREST is an academic-driven, multicenter, open-label, randomized clinical trial of fosfomycin vs ceftriaxone or meropenem (if the bacteria is ceftriaxone resistant) in the targeted treatment of bUTI caused by MDR *E coli.* Patients were recruited from June 2014 to December 2018 at 22 Spanish hospitals. The original protocol included only extended-spectrum β-lactamase (ESBL)–producing *E coli*, and the comparator was meropenem^11^; in January 2015, the protocol was modified owing to low recruitment to include any MDR *E coli*, and ceftriaxone was added as comparator for susceptible isolates.^12^ The study protocol is available in Supplement 1.

Hospitalized adult patients with monomicrobial bUTI due to *E coli* showing resistance to at least 1 drug from 3 different families to which wild-type *E coli* is susceptible^13^ and susceptibility to fosfomycin and to ceftriaxone or meropenem were eligible if deemed to need at least 4 days of intravenous therapy. Exclusion criteria were septic shock, prostatitis, kidney transplantation, polycystic kidney disease, a more than 48-hour delay in abscess drainage or obstruction release, palliative care, heart failure New Yor Heart Association (NYHA) class III or IV, liver cirrhosis, hemodialysis, allergy to study drugs, and active empirical treatment for more than 72 hours at randomization.

### Randomization and Masking

Patients were randomly assigned (1:1) to receive fosfomycin disodium (4 g every 6 hours intravenously, in 60 minutes) or a comparator: ceftriaxone (1 g every 24 hours intravenously in 2-4 minutes) or if ceftriaxone resistant, meropenem (1 g every 8 hours intravenously in 15-30 minutes). Dose adjustments for patients with kidney dysfunction are specified in the study protocol (Supplement 1). After 4 days of intravenous treatment, a switch was allowed to an in vitro active oral drug. This was oral fosfomycin trometamol 3 g every 48 hours for patients assigned to fosfomycin and cefuroxime axetil, ciprofloxacin, amoxicillin-clavulanate, or trimethoprim-sulfamethoxazole at standard dosing for patients in the comparator group, according to the susceptibility profile of the isolate. To reflect real clinical practice, patients with ceftriaxone-resistant isolates in the comparator group could also be switched to parenteral ertapenem for ambulatory treatment. The recommended total duration of treatment was 10 to 14 days. The patients were followed up for 60 days.

Assignment to the treatment group was done centrally using a previously prepared list integrated in the electronic case report form. Randomization was stratified for empirical therapy (ie, active or not) and ceftriaxone susceptibility. No blocks were used. Investigators were not blinded for drug allocation, with the exception of 2 investigators (J.S.-D. and J.R.-B.) who were blinded for checking end points.

### End Points, Study Populations, and Follow-up

The primary end point was clinical and microbiological cure (CMC) at 5 to 7 days after finalization of treatment (test of cure, TOC) in the modified intention-to-treat (MITT) population.^14^ Clinical cure was defined as resolution of all new signs and symptoms of infection at TOC; microbiological cure was defined as no isolation of the causative *E coli* strain in blood cultures from day 5 or in urine culture at TOC. Clinical failure was defined as not reaching clinical cure at TOC, worsening signs or symptoms after 48 hours of treatment, or death. Microbiological failure was defined as isolation of *E coli* in blood culture at day 5 or in urine culture at TOC.

Secondary end points included clinical and microbiological cure in the clinically evaluable population (CEP) and microbiologically evaluable population (MEP) at TOC, respectively; length of hospital stay; relapses (ie, reappearance of fever or UTI symptoms with isolation in blood or urine of *E coli* with ≤2 band differences in pulse-field gel electrophoresis [PFGE], or ≤2 drugs in susceptibility profile if not available for PFGE); reinfections (ie, the same categories as for relapses but with isolation of a different bacteria or *E coli* not fulfilling the previously mentioned criteria); 60-day mortality; and adverse events (AEs). Exploratory end points included blood levels of fosfomycin (already reported),^15^ rate of resistant bacteria isolated from follow-up cultures, and rate of ceftriaxone-resistant and carbapenem-resistant gram-negative bacteria acquisition in rectal swabs among a subset of patients.

The ITT population consisted of all randomized patients, and the MITT population consisted of patients adequately included according to study criteria who received at least 1 dose of a study drug. Exclusions from the MITT population owing to inappropriate recruitment were checked by 2 blinded investigators (J.S.-D. and J.R.-B.). The CEP included all patients evaluated at TOC or who had a previous failure. The MEP included all patients with urine cultures at TOC. Subgroup analyses were performed for age, sex, empirical treatment, Charlson Comorbidity Index score, severe sepsis status, community acquisition, and fosfomycin minimum inhibitory concentration (MIC).

### Microbiology and Rectal Carriage Substudy

Local microbiology laboratories used standard microbiological techniques for bacteria identification and susceptibility testing. Patients recruited at 3 hospitals were asked to participate in an exploratory substudy of rectal carriage by ceftriaxone-resistant or carbapenem-resistant Enterobacterales or *Acinetobacter baumannii*, using McConkey agar with cefotaxime (2 mg/L) or ChromID-ESBL (BioMérieux). Rectal swabs were taken at days 0, 3, or 4 and at end of treatment. All study isolates were sent to Hospital Universitario Virgen Macarena, where identification and antimicrobial susceptibility were confirmed using matrix-assisted laser desorption and ionization time of flight and microdilution, respectively. European Committee on Antimicrobial Susceptibility Testing recommendations^16^ were used. ESBL and carbapenemase genes were characterized by polymerase chain reaction and sequencing, and clonality of isolates was studied by PFGE.

### Data Monitoring

Collected data were verified with original data sources. Primary and secondary end points were checked for consistency by 2 blinded investigators (J.S.-D. and J.R.-B.). A data safety monitoring board reviewed the interim analysis in July 2018 and recommended continuing with recruitment but including any grade of heart failure as exclusion criteria.

### Statistical Analysis

To our knowledge, no previous trials on bUTI due to MDR *E coli* had been performed; we estimated a clinical cure rate of 85% with meropenem^17^ or ceftriaxone^12^ and 90% with fosfomycin based on our observations. To reject the inferiority of fosfomycin with a margin of −7% for CMC, 80% power and 1-sided α of 5%, 188 patients (94 patients per group) would need to be recruited. The selection of −7% as noninferiority margin was decided considering the −10% suggested by the European Medicines Agency for cUTI^18^ and given that this study included only bacteremic episodes. For the exploratory study on rectal colonization, a population of 40 patients was targeted.

The differences in proportions with 1-sided 95% CIs were calculated for categorical end points using the comparator group as reference. For secondary outcomes, analyses followed a similar approach. The treatment effect on the primary end point was also analyzed in different subgroups. Additionally, a multivariable analysis using logistic regression was performed to estimate the impact of treatment on the primary end point, including sites as random effects and other covariates showing a univariate 2-sided *P* < .20. Significance was set at *P* < .05 for comparisons not evaluating noninferiority criteria per 95% CIs. Variables not improving the model fit as assessed by using Akaike information criteria were excluded using a stepwise method. For direct comparisons between study groups, 1-sided *P* values were used. Data were analyzed using SPSS Statistics version 26 (IBM Corp) and R version 3.6.0 (R Project for Statistical Computing) in in May 2021.

## Results

### Recruitment and Patient Characteristics

Overall, 1578 patients with bacteremia due to *E coli* were screened; 161 patients were randomized, but 12 patients were found to have exclusion criteria after randomization, 5 patients withdrew consent, and 1 patient was withdrawn by the treating physician. Therefore, 143 patients composed the MITT population (Figure); 70 patients were assigned to the fosfomycin group and 73 patients to the comparator group (31 patients to ceftriaxone and 42 patients to meropenem). The CEP and MEP comprised 132 and 127 patients, respectively. Completing the recruitment was considered futile, and no additional funding was sought (see subsequent sections). Two hospitals recruited more than 20 patients each, 8 hospitals recruited 5 to 19 patients each, and 11 hospitals recruited fewer than 5 patients each.

**Figure.**
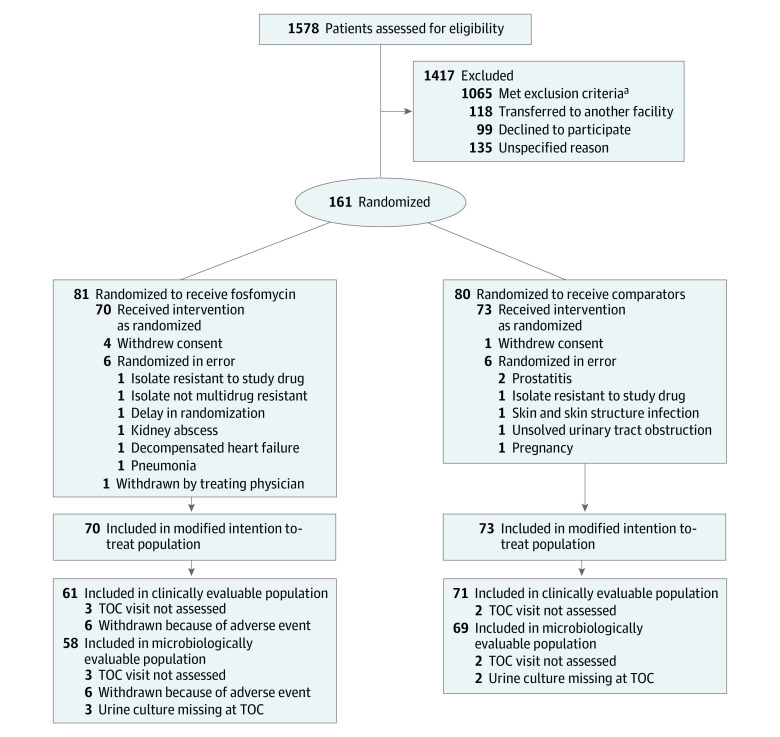
Patient Recruitment and Flow Through Study TOC indicates test of cure.

Overall, 73 patients (51.0%) were women, and their median (IQR) age was 72 (62-81) years; 100 patients (69.9%) had chronic comorbidities, and the most frequently occurring comorbidities were diabetes (38 patients [26.5%]) and cancer (30 patients [20.9%]); 45 patients (26.0%) had a urinary catheter. The characteristics of the patients by study group are shown in Table 1.^19,20,21,22^ Overall, patients in the fosfomycin and comparator groups had similar baseline characteristics (median [IQR] age, 69 [62-81] years vs 73 [62-84] years; 34 [48.6%] women vs 39 [53.4%] women), but patients in the fosfomycin group had more frequently undergone a recent invasive procedure of the urinary tract (12 patients [17.1%] vs 4 patients [5.5%]). Active empirical therapy was received by 98 patients (68.5%) overall; the mean (SD) time from blood culture extraction to randomization was 2.4 (0.6) days in the fosfomycin group and 2.4 (0.7) days in the comparator group, and the mean (SD) duration of intravenous therapy with study drugs was 5.4 (0.9) days and 5.5 (1.8) days for fosfomycin and comparators, respectively. A switch to oral therapy was performed in 60 patients (85.7%) and 48 patients (65.7%) in the fosfomycin and comparator groups, respectively; in the comparator group, 13 patients (17.8%) were switched to parenteral ertapenem. Characteristics of patients with ceftriaxone-susceptible and ceftriaxone-resistant isolates are in shown in eTable 1 and eTable 2 in Supplement 2.

**Table 1.** Baseline Characteristics of Patients in the Modified Intention-to-Treat Population^a^

Characteristic	Patients, No. (%)	
	Receiving fosfomycin (n = 70)	Receiving comparator (n = 73)
Age, median (IQR), y	69 (62-81)	73 (62-84)
Sex		
Women	34 (48.6)	39 (53.4)
Men	36 (51.4)	34 (46.6)
Charlson Comorbidity Index score^b^		
Median (IQR)	1 (0-3)	2 (1-3)
≥3	22 (31.4)	22 (30.1)
Congestive heart failure^c^	8 (11.4)	11 (15.1)
Chronic pulmonary disease^c^	12 (17.1)	11 (15.1)
Diabetes^c^	19 (27.1)	19 (26.0)
Chronic kidney disease^c^	9 (12.9)	14 (19.2)
Cancer^c^	14 (20.0)	16 (21.9)
Full dependence for basic activities	4 (5.7)	6 (8.2)
Urinary catheter at enrollment	21 (30.0)	22 (30.1)
Invasive procedure in the urinary tract in previous month^d^	12 (17.1)	4 (5.5)
Immunosuppressive drugs	7 (10.0)	9 (12.3)
Present infection		
Community-acquired infection^e^	33 (47.1)	39 (53.4)
Health care–associated infection^e^	25 (35.7)	23 (31.5)
Nosocomial infection^e^	12 (17.1)	11 (15.1)
Low urinary tract symptoms^f^	39 (55.7)	45 (61.6)
Flank pain or tenderness	27 (38.6)	26 (35.6)
Severe sepsis at presentation^g^	15 (21.4)	22 (30.1)
Pitt score, median (IQR)^h^	1 (0-1.25)	1 (0-2)
eGFR<60 mL/min/1.73 m^2^ at enrollment	21 (30.0)	22 (30.1)
Hydronephrosis in echography at enrollment	9 (12.9)	6 (8.2)
Active treatment ≤24 h after blood culture	48 (68.6)	50 (68.5)
Time until active treatment, mean (SD), d	0.9 (1.2)	0.9 (1.1)
Time until randomization, mean (SD), d	2.4 (0.6)	2.4 (0.7)
Removal or change of urinary catheter ≤48 h after enrollment^i^	17/21 (80.9)	19/22 (86.3)
Susceptibility of baseline *Escherichia coli* (local laboratory)		
Amoxicillin	7 (10)	5 (6.8)
Amoxicillin-clavulanic acid	38 (54.3)	29 (39.7)
Piperacillin-tazobactam	55 (78.6)	54 (74.0)
Cefotaxime	32 (45.7)	33 (45.2)
Cefepime	34 (48.6)	32 (48.6)
Meropenem	70 (100)	73 (100)
Ciprofloxacin	14 (20.0)	11 (15.1)
Trimethoprim-sulfamethoxazole	33 (47.1)	21 (28.8)
Amikacin	59 (84.3)	66 (90.4)
Fosfomycin	70 (100)	73 (100)
Length of intravenous therapy with study drug, mean (SD), d	5.4 (0.9)	5.5 (1.8)
Length of antibiotic therapy with study drug, mean (SD), d	11.5 (3.9)	11.9 (2.0)
Oral antibiotic therapy after intravenous therapy with study drug	60 (85.7)	48 (65.7)
Oral drug used		
Fosfomycin trometamol	60 (85.7)	1 (1.4)^j^
Cefuroxime axetil	0	28 (38.3)
Amoxicillin-clavulanic acid	0	7 (9.6)
Trimethoprim-sulfamethoxazole	0	7 (9.6)
Ciprofloxacin	0	5 (6.8)
Parenteral ertapenem after study drug	0	13 (17.8)

Abbreviation: eGFR, estimated glomerular filtration rate.

^a^ Data are expressed as No. (%) of participants unless otherwise indicated.

^b^ Provides a 10-year mortality risk based on weighted comorbid conditions, ranging from 0 to 29, with a score of 4 associated with an estimated 10-year survival of 53%.^19^

^c^ These variables were assessed at enrollment by site investigators based on definitions in the Charlson Comorbidity Index.

^d^ Included open surgical treatment of the urinary tract, nephrostomy, double jack catheter placement, cystoscopy, transurethral resection, and transrectal prostate biopsy.

^e^ According to Friedman criteria.^20^ In summary, nosocomial infection is defined as occurring among patients hospitalized for 48 hours or more; health care–associated infection is defined as occurring among patients who received intravenous therapy, specialized nursing care at home in the 30 days before the bloodstream infection for which the patient was recruited, attended a hospital or hemodialysis clinic or received intravenous chemotherapy in the 30 days before the infection, was hospitalized in an acute care hospital for 2 or more days in the 90 days before the infection, or resided in a nursing home or long-term care facility; and community-acquired infection is defined as those not fulfilling the criteria for nosocomial or health care–associated infection.

^f^ Included dysuria, urinary frequency or urgency, and suprapubic pain.

^g^ Defined according to the 2001 Society of Critical Care Medicina/European Society of Intensive Care Medicine/American College of Clinical Pharmacology American Thoracic Society/Surgical Infection Society International Sepsis Definitions Conference.^21^

^h^ Provides a measure of in-hospital mortality risk among patients with bloodstream infections based on clinical variables, ranging from 0 to 14, with a Pitt score of 4 or more associated with a risk of mortality of approximately 40%.^22^

^i^ The denominators are the number of patients with a urinary catheter.

^j^ One patient received fosfomycin trometamol by mistake.

Isolates from 68 of 81 patients with ceftriaxone-resistant *E coli* were available for further studies; 64 isolates (94.1%) were ESBL producers; the most frequent ESBLs were CTX-M-15 (38 isolates [59.3%]) and CTX-M-14 (13 isolates [20.3%]) (eTable 3 in Supplement 2).

### Primary Analysis

CMC rates in the MITT population were 48 patients (68.6%) in the fosfomycin group and 57 patients (78.1%) in the comparator group (difference, −9.4 percentage points; 1-sided 95% CI, −21.5 to ∞ percentage points; *P* = .10) (Table 2). Continuing the recruitment until completing the calculated sample size was considered futile because to demonstrate noninferiority, all 24 pending patients to be assigned to fosfomycin but 11 of 21 pending patients (52.3%) to be assigned to the comparators would need to achieve CMC. Therefore, fosfomycin was determined to have not reached noninferiority criteria.

**Table 2.** Patients Reaching CMC and Reasons for Not Reaching It

	Patients, No./total No. (%)		Risk difference (1-sided 95% CI)^a^	*P* value, 1-sided
	Receiving fosfomycin	Receiving comparator		
**CMC at TOC among MITT (measures of success)**				
All patients	48/70 (68.6)	57/73 (78.0)	−9.4 (−21.5 to ∞)	.10
Patients with ceftriaxone-susceptible isolates^b^	25/31 (80.6)	27/31 (87.0)	−6.4 (−21.7 to ∞)	.24
Patients with ceftriaxone-resistant isolates^b^	23/39 (59.0)	30/42 (71.4)	−12.4 (−29.8 to ∞)	.12
**Reasons for not reaching CMC at TOC among MITT (measures of failure)**				
Clinical or microbiological failure				
All patients	10/70 (14.3)	14/73 (19.7)	−5.4 (−∞ to 4.9)	.19
Patients with ceftriaxone-susceptible isolates^b^	3/31 (9.7)	4/31 (12.9)	−3.2 (−∞ to 10.0)	.34
Patients with ceftriaxone-resistant isolates^b^	7/39 (17.9)	10/42 (23.8)	−8.9 (−∞ to 6.9)	.25
Other reasons				
Withdrawn because of adverse events	6/70 (8.5)^c^	0/73 (0)	8.5 (−∞ to 13.9)	.006
Missed assessment at TOC	3/70 (4.2)	2/73 (2.7)	1.5 (−∞ to 6.5)	.31
TOC assessed but urine culture at TOC not available	3/70 (4.2)	0/73 (0)^d^	4.2 (−∞ to 8.1)	.03

Abbreviations: CMC, clinical and microbiological cure; MITT, modified intention-to-treat population; TOC, test of cure.

^a^ The risk difference was calculated with a 1-sided 95% CI. The margin for noninferiority was set at −7%. The lower bound of the CI for the primary end point (ie, CMC at TOC in the MITT) exceeded this threshold in the primary analysis population, thus excluding noninferiority.

^b^ The comparators for ceftriaxone-susceptible and ceftriaxone-resistant isolates were ceftriaxone and meropenem, respectively.

^c^ Heart failure occurred among 4 patients, rash among 1 patient (who also had heart failure), cholecystitis among 1 patient, and persistence of fever (later assigned to cancer) among 1 patient.

^d^ There were 2 patients with urine culture missing at TOC, but they also had clinical failure and therefore they were classified as having clinical or microbiological failure in this table.

Reasons for not reaching CMC are specified in Table 2. Clinical or microbiological failure was numerically lower with fosfomycin (10 patients [14.3%] vs 14 patients [19.7%]; difference, −5.4 percentage points; 1-sided 95% CI, −∞ to 4.9 percentage points; *P* = .19). Other reasons were more frequent in the fosfomycin group; specifically, discontinuation because of adverse events occurred among 6 patients (8.5%) treated with fosfomycin and no patients treated with comparators (*P* = .006). Adverse events leading to fosfomycin discontinuation were heart failure among 4 patients (5.7%) and alithiasic cholecystitis and persistent fever among 1 patient each (1.4%) (eTable 4 in Supplement 2).

### Secondary Outcomes

Clinical cure in the CEP was more frequent among patients treated with fosfomycin than among patients treated with comparators (59 of 61 patients [96.7%] vs 64 of 71 patients [90·1%]; difference, 6.6 percentage points; 1-sided 95% CI, −0.2 to ∞ percentage points; *P* = .05). Microbiological cure in the MEP occurred among 48 of 58 patients (82.8%) treated with fosfomycin and 59 of 69 patients (85.5%) treated with comparators (difference, −2.7 percentage points; 1-sided 95% CI, −13.3 to ∞ percentage points; *P* = .33). In the CEP, relapse occurred among 8 patients (13.1%) treated with fosfomycin and 6 patients (8.4%) treated with comparators, respectively; reinfection rates were similar in the 2 study groups. Crude mortality in the CEP occurred among 2 patients [3.3%] treated with fosfomycin and 2 patients [2.8%] treated with comparators. Mean (SD) length of hospital stay after randomization was 7.8 (8.0) days in the fosfomycin group and 6.4 (4.7) days in the comparator group (Table 3).

**Table 3.** Analysis of Secondary End Points

	Patients, No./total No. (%)^a^		Risk difference (1-sided 95% CI)^b^	*P* value, 1-sided
	Receiving fosfomycin	Receiving comparators		
**Measure of success**				
Clinical cure at TOC (CEP)				
All patients	59/61 (96.7)	64/71 (90.1)	6.6 (−0.2 to ∞)	.05
Patients with ceftriaxone-susceptible isolates	29/29 (100)	29/31 (93.5)	6.5 (−1.1 to ∞)	.08
Patients with ceftriaxone-resistant isolates	30/32 (93.8)	35/40 (87.5)	6.3 (−5.2 to ∞)	.18
Microbiological cure at TOC (MEP)				
All patients^c^	48/58 (82.8)	59/69 (85.5)	−2.7 (−13.3 to ∞)	.33
Patients with ceftriaxone-susceptible isolates	25/28 (89.3)	29/31 (93.5)	−4.2 (−18.4 to ∞)	.28
Patients with ceftriaxone-resistant isolates	23/30 (76.6)	30/38 (78.9)	−2.3 (−18.9 to ∞)	.41
**Measure of failure**				
30-day mortality (CEP)				
All patients	2/61 (3.2)	2/71 (2.8)	0.4 (−∞ to 5.2)	.44
Patients with ceftriaxone-susceptible isolates	1/29 (3.4)	0/31 (0)	3.3 (−∞ to 8.8)	.15
Patients with ceftriaxone-resistant isolates	1/32 (3.1)	2/40 (5.0)	−1.9 (−∞ to 5.8)	.34
Relapse (CEP)				
All patients	8/61 (13.1)	6/71 (8.4)	4.7 (−∞ to 13.5)	.19
Patients with ceftriaxone-susceptible isolates	3/29 (10.3)	1/31 (3.2)	7.1 (−∞ to 17.6)	.13
Patients with ceftriaxone-resistant isolates	5/32 (15.6)	5/40 (12.5)	3.1 (−∞ to 16.5)	.35
Reinfection (CEP)				
All patients	4/61 (6.5)	4/71 (5.6)	0.9 (−∞ to 7.7)	.41
Patients with ceftriaxone-susceptible isolates	1/29 (3.4)	1/31 (3.2)	0.2 (−∞ to 7.7)	.48
Patients with ceftriaxone-resistant isolates	3/32 (9.3)	3/40 (7.5)	1.8 (−∞ to 12.5)	.39
**Other measure**				
Hospitalization after randomization, mean (SD), d				
All patients	7.8 (8.0)	6.4 (4.7)	1.4 (−∞ to 3.1)	.10
Patients with ceftriaxone-susceptible isolates	6.0 (1.9)	4.4 (1.3)	1.6 (−∞ to 2.2)	<.001
Patients with ceftriaxone-resistant isolates	9.5 (10.8)	7.9 (5.8)	2.9 (−∞ to 6.1)	.07

Abbreviations: CEP, clinically evaluable population; MEP, microbiologically evaluable population; TOC, test of cure.

^a^ The comparators for ceftriaxone-susceptible and ceftriaxone-resistant isolates were ceftriaxone and meropenem, respectively. For each end point, the appropriate population is specified.

^b^ The risk difference was calculated with a 1-sided 95% CI.

^c^ All microbiological failures were due to positive urine cultures only.

### Subgroup Analyses and Multivariate Analysis

The fosfomycin group had decreased CMC rates in all subgroups except among patients with severe sepsis, among whom 13 of 15 patients in the fosfomycin group (86.7%) and 16 of 22 patients in the comparator group (72.7%) achieved CMC (difference, 14.0 percentage points; 1-sided 95% CI, −8.6 to ∞ percentage points; *P* = .15) (Table 4). Regarding clinical or microbiological failure, fosfomycin had decreased rates in all subgroups (eTable 5 in Supplement 2). Outcomes were also analyzed among patients who switched to oral drugs (or parenteral ertapenem in the comparator group). Among them, CMC was achieved among 48 of 60 patients (80.0%) treated with fosfomycin who switched to fosfomycin trometamol and among 47 of 61 patients (77.0%) treated with comparators who switched to oral drugs or parenteral ertapenem (1-sided *P* = .34); relapse occurred among 8 patients (13.3%) and 4 patients (8.1%), respectively (*P* = .17) (eTable 6 in Supplement 2).

**Table 4.** Analyses of Clinical and Microbiological Cure Rates at the Test of Cure in Subgroups of Modified Intention-to-Treat Population

Subgroup	Patients, No./total No. (%)		Risk difference (1-sided 95% CI)^a^	*P* value, 1-sided
	Receiving fosfomycin	Receiving comparator		
Age, y				
≤80	34/50 (68.0)	40/53 (75.5)	−7.5 (−22.0 to ∞)	.19
>80	14/20 (70.0)	17/20 (85.0)	−15.0 (−36.7 to ∞)	.12
Women	24/34 (70.6)	29/39 (74.4)	−3.8 (−21.0 to ∞)	.35
Men	24/36 (66.7)	28/34 (82.4)	−15.7 (−32.8 to ∞)	.06
Empirical treatment				
Active	32/48 (66.7)	37/50 (74.0)	−7.3 (−22.5 to ∞)	.21
Inactive	16/22 (72.7)	20/23 (87.0)	−14.3 (−34.2 to ∞)	.11
Charlson Comorbidity Index score^b^				
≤2	33/48 (68.8)	41/51 (80.4)	−11.6 (−25.9 to ∞)	.09
>2	15/22 (68.2)	16/22 (72.7)	−4.5 (−27.1 to ∞)	.37
Severe sepsis^b^				
No	35/55 (63.6)	41/51 (80.4)	−16.8 (−31.2 to ∞)	.02
Yes	13/15 (86.7)	16/22 (72.7)	14.0 (−8.6 to ∞)	.15
Community-acquired infection^b^				
Yes	22/33 (66.7)	29/39 (74.4)	−7.7 (−25.3 to ∞)	.23
No	26/37 (70.3)	28/34 (82.4)	−12.1 (−28.7 to ∞)	.11
Fosfomycin MIC, mg/L^c^				
≤1	19/27 (70.4)	17/20 (85.0)	−14.6 (−35.1 to ∞)	.12
>1	22/33 (66.7)	28/37 (75.7)	−9.0 (−26.7 to ∞)	.20

Abbreviation: MIC, minimum inhibitory concentration.

^a^ The risk difference was calculated with a 1-sided 95% CI.

^b^ For definitions, see Table 1.

^c^ MIC was studied by agar microdilution in 117 available isolates.

Multivariate analysis was performed to assess the effect of treatment group on CMC, controlling for residual imbalances in exposures. The nonadjusted odds ratio (OR) for CMC among patients receiving fosfomycin, vs patients receiving comparators, was 0.61 (95% CI, 0.28-1.29; *P* = .20), and after adjustment by other covariates, the OR was 0.55 (95% CI, 0.24-1.21; *P* = .14) (eTable 7 in Supplement 2)

### Safety

AEs were reported among 44 patients (62.9%) and 41 patients (56.2%) in the fosfomycin and comparator groups, respectively (*P* = .41). Serious AEs were reported among 13 patients treated with fosfomycin (18.6%) and 10 patients treated with comparators (13.7%) (*P* = .42). Details are shown in eTable 8 and eTable 9 in Supplement 2. In the fosfomycin group, 6 patients (8.6%) developed heart failure (1 patient had 2 episodes, with the second episode occurring after the drug had been discontinued); all these patients were aged 81 years or older, 2 had chronic heart failure, and 3 had chronic kidney insufficiency. Among 5 of these patients, heart failure was considered serious, and among those 5 patients, the drug was discontinued among 4.

### Microbiological Studies

Considering all positive urine cultures obtained after treatment and until the end of follow-up (ie, at TOC, end of follow-up, and unscheduled visits), ceftriaxone-resistant bacteria were isolated among 20 patients (29.5%) treated with fosfomycin and 27 patients (36.9%) treated with comparators (*P* = .29. Meropenem-resistant bacteria were isolated among 2 patients (2.8%) and 3 patients (4.1%), respectively (*P* > .99), and fosfomycin-resistant bacteria were isolated among 8 patients (11.4%) and 6 patients (8.2%), respectively (*P* = .58) (eTable 10 in Supplement 2).

In the rectal colonization substudy, 38 patients were included; 0 of 21 patients treated with fosfomycin and 4 of 17 patients (23.5%) treated with a comparator acquired a new ceftriaxone-resistant or meropenem-resistant gram-negative bacterial infection (1-sided *P* = .01). Among the latter, 2 patients treated with ceftriaxone acquired ESBL-producing *E coli* and *Klebsiella pneumoniae*, and 2 treated with meropenem acquired an OXA-48–producing *K. pneumoniae* and *Acinetobacter baumannii*.

## Discussion

In this randomized clinical trial, fosfomycin did not reach the noninferiority criteria in the treatment of bUTI due to MDR *E coli*. However, this was not due to lack of efficacy; in fact, the clinical and microbiological failure rate was numerically lower with fosfomycin in the MITT, for which the 1-sided 95% CI of the difference was the below the −7% noninferiority margin. The high success rate with fosfomycin among patients with severe sepsis reinforces the idea that fosfomycin is efficacious in this infection.

Previous randomized clinical trials on intravenous fosfomycin mostly included nonbacteremic cUTI. A randomized clinical trial in Sweden^23^ included 38 adults with pyelonephritis (including 30 patients infected by *E coli*), treated with fosfomycin (2 g every 8 hours) or ampicillin (2 g every 8 hours) and found 44% and 27% clinical cure rates. A phase 2/3 double-blind randomized clinical trial^8^ compared fosfomycin (6 g every 8 hours) with piperacillin-tazobactam (4.5 g every 8 hours) among patients with cUTI; 73% were caused by *E coli*, and 9% were bacteremic. CMC was reached among 64.7% of patients with fosfomycin and 54.5% of patients with piperacillin-tazobactam. The patients in that study were younger and more frequently women than in our study.

Fosfomycin was discontinued among 6 patients because of AEs in our study. This was not the case in the previously mentioned double-blind trial^8^ using a similar total daily dose, suggesting a negative impact of the open design against fosfomycin. Nevertheless, heart failure was reported among 6 patients treated with fosfomycin; all but 1 had chronic heart failure (NYHA class I or II) or kidney insufficiency, and all were older than age 80 years. This AE was not described in the cUTI trial,^8^ which might be because of the participants’ difference in age, and was described among 2 of 2672 patients in a meta-analysis.^6^ Heart insufficiency may be caused by the sodium content (14.4 mEq/g) of the intravenous formulation. We suggest avoiding intravenous fosfomycin among patients aged older than 80 years and those with chronic heart or kidney insufficiency. Hypokalemia, usually mild, is a well-known AE associated with fosfomycin.^6,8^

The fosfomycin dose used in our study was chosen based on pharmacodynamic data,^24^ allowing a 90% probability of target attainment for bactericidal effect for an MIC of 32 mg/L or less.^15^ However, selection of resistant subpopulations is a concern; whether it can be avoided by using other dosing regimens is unclear.^23,25,26^ In our study, clinical failure due to development of fosfomycin-resistant *E coli* during treatment did not occur.

To the best of our knowledge, this is the first trial to include fosfomycin trometamol as an oral switch among patients with bacteremic infections; its concentrations are high in urine but low in plasma. However, bacteremia in bUTI is an epiphenomenon, and once the parenchymal component of the infection is controlled, urine concentrations may be more important. The outcome data from the subgroup analyses among patients who were switched were encouraging. The investigation of the ecologic impact of the study drugs was exploratory. Overall, the data obtained support the idea that fosfomycin may cause less ecological damage than ceftriaxone and meropenem, and the findings may open the door to further studies.

### Limitations and Strengths

This study has several limitations. The calculated sample size was not reached. Additionally, a highly exigent noninferiority margin was chosen. Despite end points being checked by blinded investigators, a lack of blinding may have influenced the delay of hospital discharge and withdrawal of some patients treated with fosfomycin. The options for switching were diverse in the comparator group to mimic standard practice, because the susceptibility of the isolates is unpredictable; however, their efficacy is similar. The rectal colonization study was performed among a small subset of patients.

Some strengths beyond randomization include the pragmatic design, monitoring of quality of data, recruitment of older patients with comorbidities, and exclusion of patients stable enough to allow an early discharge with oral drugs. Additionally, exploratory data on the ecological impact of the study drugs were provided.

## Conclusions

Fosfomycin did not demonstrate noninferiority in the treatment of bUTI caused by MDR *E coli*. However, the data suggest that the drug is effective and may be considered among selected patients, particularly those without previous heart disease and with low risk of sodium overload–related problems. Some safety concerns with fosfomycin were raised. The potential decreased ecological impact of fosfomycin deserves further study.

## Notes

Supplement 1.
Trial Protocol

Supplement 2. **eTable 1.** Baseline Characteristics of Patients in Modified Intention-to-Treat Population With Ceftriaxone-Susceptible Isolates by Fosfomycin vs Ceftriaxone **eTable 2.** Baseline Characteristics of Patients in Modified Intention-to-Treat Population With Ceftriaxone-Resistant Isolates by Fosfomycin vs Meropenem **eTable 3.** Extended-Spectrum β-Lactamases Produced by Ceftriaxone-Resistant Isolates at Baseline **eTable 4.** Patients Who Did Not Reach Clinical and Microbiological Cure at Test of Cure for Reasons Other Than Clinical or Microbiological Failure **eTable 5.** Analyses of Clinical or Microbiological Failure Rates at Test of Cure in Subgroups of Modified Intention-to-Treat Population **eTable 6.** Clinical and Microbiological Cure and Relapse Rates Among Patients Who Switched to Oral Drugs or Parenteral Ertapenem **eTable 7.** Multivariate Analysis of Variables Associated With Clinical and Microbiological Cure in Modified Intention-to-Treat Population **eTable 8.** Adverse Events Reported **eTable 9.** Description of Serious Adverse Events **eTable 10.** Ceftriaxone-Resistant Bacteria Isolated After Treatment

Supplement 3.
Nonauthor Collaborators

Supplement 4.
Data Sharing Statement
