# Supplementary material for: Effectiveness of Fosfomycin for the Treatment of Multidrug-Resistant Escherichia coli Bacteremic Urinary Tract Infections: A Randomized Clinical Trial
Source: JAMA Netw Open. 2022 Jan 13;5(1):e2137277. doi: 10.1001/jamanetworkopen.2021.37277 (PMC8759008; doi:10.1001/jamanetworkopen.2021.37277)
Supplement: Supplement 1. — Trial Protocol [file jamanetwopen-e2137277-s001.pdf]

CLINICAL TRIAL PROTOCOL

RANDOMIZED, MULTICENTER, OPEN LABEL, CONTROLLED, FASE III CLINICAL TRIAL TO EVALUATE THE EFFICACY OF FOSFOMYCIN VS MEROPENEM OR CEFTRIAXONA IN THE TARGETED TREATMENT OF BACTEREMIC URINARY TRACT INFECTION CAUSED BY MULTIDRUG-RESISTANT *ESCHERICHIA COLI*

CÓDE: FOREST

Nº EUDRACT: 2013-002922-21

VERSION 4.0 (27 September 2018)

1.- GENERAL INFORMATION

1.1 Trial Identification

Code: FOREST
EudraCT: 2013-002922-21

1.2 Type of trial

Phase III, open label, controlled, randomized, multicenter

1.3 Promoter

Fundación Investigación Sevilla (FISEVI).
Hospital Universitario Virgen del Rocío, Edificio de laboratorios, 6ª planta, Avda. Manuel Siruot S/N, 41013, Sevilla, Spain
Tel. 955013645 / 955013284 (sede Macarena)

1.3.1 Authorized person by the promoter

Clara M. Rosso Fernández
Unidad de Investigación Clínica y Ensayos Clínicos, Hospital Universitario Virgen del Rocío, Avda. Manuel Siruot S/N, 41013, Sevilla, Spain
Tel. 955013645 / 955013284 (sede Macarena)

1.3.2 Monitorization

Unidad de Investigación Clínica y Ensayos Clínicos, Hospital Universitario Virgen del Rocío, Avda. Manuel Siruot S/N, 41013, Sevilla, Spain
Tel.:955313414

1.4 Principal investigator

Jesús Rodríguez Baño
Servicio de Enfermedades Infecciosas, Hospital Universitario Virgen Macarena, Avda. Dr Fedriani 3, 41009, Sevilla, Spain

1.5 Description of study products

Experimental treatment: Fosfomycin disodiium

Control treatment 1: Meropenem

Control treatment 2: Ceftriaxona

2.- JUSTIFICATION

Enterobacteriaceae (and particularly *Escherichia coli*) that produce extended spectrum beta-lactamases (ESBL), plasmid AmpCs, or are resistant to quinolones by chromosomal mechanisms, have become a frequent cause of infection worldwide in the last decade [1]. This fact is of great importance for two main reasons: on the one hand, these are infections associated with increased mortality and hospital stay [2], and on the other, it is common for these bacteria to be multidrug-resistant [3], which has forced to modify treatment protocols in most health centers. In this sense, given that cephalosporins are the drugs of choice for strains resistant to quinolones, and carbapenems for serious infections caused by strains resistant to cephalosporins and quinolones, the consequence is that the increase in the consumption of these drugs is probably contributing to the selection of Gram negative rods resistant to cephalosporins in the first case and carbapenems in the second, and therefore facilitating the rapid dissemination of ESBL and carbapenemase-producing enterobacteria [4]. In this context, finding therapeutic alternatives to cephalosporins and carbapenems for the treatment of multidrug-resistant Enterobacteriaceae is an urgent need; if these alternatives were also useful for the treatment of carbapenemase-resistant Enterobacteriaceae, even better. The most plausible alternatives are beta-lactamase inhibitors, temocillin (not available in Spain), aminoglycosides (whose limitations are known [5]) and fosfomycin.

Fosfomycin is an antimicrobial discovered more than 40 years ago that acts at the level of bacterial cell wall biosynthesis through the inhibition of peptidoglycan formation. This antibiotic maintains excellent in vitro activity against multidrug-resistant and extremely resistant Enterobacteriaceae [10], and specifically against ESBL-producing E. coli [11]. Although it is true that the dissemination of resistant strains has been described in our environment, which has been related to the increase in the consumption of the drug for the oral treatment of uncomplicated urinary infections [12], it seems that fosfomycin-resistant Enterobacteriaceae generally present a lower fitness than the sensitive ones, which will have allowed the maintenance of their activity over time [13]. Fosfomycin, in its formulation for intravenous administration (fosfomycin disodium) is approved in Spain, according to its technical file, for use in a wide variety of infections, including complicated or serious urinary infections and septicemias, produced by sensitive microorganisms; in the case of "septicemia", the label recommends combined treatment. The recommended dose is 4 grams every 6-8 hours. It is important to consider that when this drug was developed, the requirement of the regulatory agencies was different from the current one; probably this fact and its subsequent disuse due to the availability of other drugs have motivated that clinical studies with this drug are truly scarce.

In 2010, a systematic review was published on the efficacy of fosfomycin in the treatment of infections caused by multidrug-resistant enterobacteria [14]; This study showed that the existing information on its efficacy in systemic infections caused by these microorganisms was practically non-existent, and was limited to some small series of cases in which it had been used in combination with other active drugs. Subsequently, not much more information has been generated. The preliminary results

of a systematic review that is being carried out by the research team responsible for this study (registered at http://www.crd.york.ac.uk/PROSPERO), precisely to develop this project, indicate It is clear that there are no adequate studies in which fosfomycin has been compared with the reference drugs and that meet current requirements neither for invasive infections in general nor for upper urinary tract infections caused by ESBL-producing enterobacteriaceae (and with other resistance mechanisms) in particular.

In the current situation of lack of new antimicrobials and bacterial resistance, fosfomycin has been identified as one of the orphan antibiotics of high potential value that would be of great interest to investigate [15]. After reviewing clinical trial records, no ongoing studies with fosfomycin for the proposed or similar indication have been found. Therefore, there is an opportunity to design and conduct randomized clinical trials to evaluate its efficacy and safety. In this sense, it seems to us especially important to consider the new paradigms proposed for the design of randomized trials with antibiotics for the treatment of resistant bacteria, since they allow increasing their efficiency and real utility to respond to existing clinical needs, and which include the conducting trials for targeted therapy and studies of pharmacokinetic and pharmacodynamic parameters (PK/PD) [16,17].

The main concern with the use of fosfomycin is the possibility of development of resistance during treatment. The results of a recent systematic review of the literature suggest that this risk is much lower for *E. coli* than for other Enterobacteriaceae, *Pseudomonas aeruginosa* or *Staphylococcus aureus* [13]. Urinary origin, especially if there is no obstruction of the urinary tract, is the one that is associated with lower mortality in patients with bacteremia [18]; likewise, the development of resistance in the course of treatment is less likely in this infection [13]. Therefore, the investigation of its efficacy in monotherapy in urinary infections due to *E. coli*, at least if an adequate control of the focus has been carried out if necessary, is justified.

The few PK/PD studies carried out indicate that after an intravenous dose of 4 grams, plasma concentrations are reached sufficient for the treatment of systemic infections by susceptible microorganisms for at least 4 hours [19, 20], and since it is excreted unchanged by urine, its urinary concentrations are also adequate for these infections [19]. Regarding the oral formulation of fosfomycin trometamol, its plasma concentrations are low, but the urinary ones are very high [20]; has been shown to be useful in observational studies for the treatment of cystitis and urinary tract infections complicated by ESBL-producing E. coli [14,21] and therefore its use for the sequential treatment of invasive urinary tract infections is reasonable, once the bacteremia and the focus of infection. Furthermore, it has recently been shown to reach high concentrations in the prostate [22].

Although the FDA considers “complicated urinary tract infection” as a syndrome to be evaluated for new therapeutic alternatives [23], we consider this to be an extremely heterogeneous syndrome in the FDA definition, since it includes from asymptomatic bacteriuria in certain patients, lower urinary tract infection in catheterized patients, or pyelonephritis in patients with urinary tract disorders. In the opinion of the research group responsible for this trial, the investigation of fosfomycin in this syndrome would

contribute little in terms of its real utility, and would not provide adequate data for its use in patients with bacteremia. On the contrary, the study in well-selected patients with bacteremic urinary tract infections using adequate diagnostic criteria, responds to a real clinical need. Also, the results will be easily extrapolated to non-bacteremic urinary tract infections provided the circumstances contemplated in the proposed trial are met. The treatment chosen for the control group ("standard treatment") is ceftriaxone, in the case of infection by strains resistant to quinolones and sensitive to cephalosporins (recommended in the IDSA guideline at a dose of 1 g per day), and meropenem for those resistant to cephalosporins. Other options, but some centers do not have these drugs in their pharmacotherapeutic guidelines for the treatment of urinary tract infections.

Regarding the alternatives for sequential treatment in the arms of the control group, an attempt has been made to reflect clinical practice, so that several drugs are prioritized according to in vitro activity. In the case of strains resistant to quinolones but sensitive to cephalosporins, oral cefuroxime axetil is considered, and if the patient does not tolerate the oral route for some reason, intramuscular ceftriaxone. In the case of strains resistant to cephalosporins, the oral drugs with the most experience in treating these infections are fluoroquinolones; This is why they are in the first place, although they can rarely be used since most strains of *E. coli* ESBL are resistant. Second, we include amoxicillin / clavulanate given the published experience in this regard in these strains, and that between 40% and 60% may be sensitive [8,21]. Third, trimethoprim-sulfamethoxazole is included due to the experience in pyelonephritis with this drug, although most of these bacteria are also resistant. Finally, the use of parenteral ertapenem or amikacin is allowed, since this allows outpatient treatment in patients without oral treatment options.

Finally, regarding the safety of fosfomycin disodium, the high sodium content of the intravenous compound should be considered (330 mg of sodium per gram of fosfomycin), which should be taken into account in patients requiring sodium restriction, as stated in The datasheet. Apart from this, the existing data indicate that it is generally a well tolerated drug [14], but it is clear that more data collected from well designed and conducted studies is needed. Therefore, the present clinical trial is considered as an initial step in the investigation of an orphan antimicrobial, of low cost and great potential, as a therapeutic alternative for a frequent infection and in well-selected patients. These results may have a great impact on the use of antibiotics and on the development of new projects with this drug, both in monotherapy and in combination therapy.

3.- HYPOTHESIS AND OBJETIVES

3.1 Hypothesis

Targeted treatment of multidrug-resistant *Escherichia coli* bacteremic urinary tract infection with intravenous fosfomycin disodium is not inferior in clinical efficacy to treatment with meropenem or ceftriaxone, in selected patients, and may therefore represent a therapeutic alternative in these infections that makes it possible to reduce the use of carbapenems and cephalosporins.

3.2 Objetives

Main

To demonstrate the non-inferiority in terms of efficacy of intravenous fosfomycin compared to meropenem or ceftriaxone in the targeted treatment of multidrug-resistant *E. coli* bacteremia, measured as clinical and microbiological cure at 5-7 days after the end of treatment (test of cure).

Secondary

To assess the early clinical and microbiological response (day 5-7 of treatment) in the cure test in both arms.

To compare the mortality and hospital stay in the first 30 days of patients treated with fosfomycin and meropenem or ceftriaxone.

To assess the safety of intravenous fosfomycin in this indication.

To compare the frequency of recurrences (relapses and reinfections) in both arms.

To study the pharmacokinetics of intravenous fosfomycin in patients with bacteremic urinary infection due to multidrug-resistant *E. coli*.

To compare the impact of fosfomycin and comparators in the colonization by multidrug-resistant Gram negative bacilli.

To evaluate the emergence of fosfomycin-resistant isolates during treatment

4.- VARIABLES AND DESIGN

4.1 Variables

4.1.1 Primary endpoint

Clinical and microbiological cure at test of cure (5-7 days after end of treatment).

Clinical cure is defined as the resolution of all the symptoms of bacteremia and urinary tract infection that were present at the time of the initial blood culture collection. Specifically, clinical cure includes resolution of the following key symptoms /signs, if present: fever (axillary T> 38ºC), lumbar pain, renal fist-percussion, dysuria, frequency, urinary urgency, and suprapubic pain. In patients probed prior to the onset of the current infection, the resolution of the local symptoms that led to their placement will not be required, considering in this case a return to the baseline situation as a cure. In elderly patients (> 70 years) in whom the main manifestation is cognitive impairment in the absence of other key manifestations of urinary tract infection, a return to the previous cognitive situation will be considered a clinical cure.

Microbiological cure requires the disappearance of the initial bacteremia at test of cure. Furthermore, in the cases in which there is an initial positive urine culture with the same strain as that isolated in blood (taken at visit 1 or in the 96 hours prior to inclusion in the study), it will be required to be negative.

4.1.2 Secondary endpoints

Early clinical cure. Early clinical cure is defined in a similar way to that explained above for clinical cure at the cure visit, assessed after 5 full days of treatment (visit 3).

Early microbiological cure. Early microbiological cure requires the disappearance of the initial bacteremia, that is, a negative blood culture after five full days of treatment (visit 3) or earlier (visit 2). Furthermore, in cases where there is an initial positive urine culture with the same strain as that isolated in blood, it will be required to be negative.

Recurrences (relapses and reinfections) in the first 60 days.

Recurrence is defined as the reappearance of symptoms of urinary tract infection together with a positive blood culture or urine culture for the same microorganism isolated in the initial blood culture in a patient with clinical and microbiological cure at the visit duration (5-7 days after completion of treatment).

Reinfection is defined in a similar way, but with isolation of a strain other than the initial one. Symptoms of urinary tract infection are fever (axillary T> 38ºC), lumbar pain, positive renal fist-percussion, dysuria, frequency, urgency, urgency and suprapubic pain. In elderly patients (> 70 years), altered baseline cognitive status with no other explanatory cause may be considered a symptom of urinary infection.

Mortality. Mortality is considered in the first 30 days and hospital stay.

Frequency and severity of adverse effects.

Plasma concentrations of fosfomycin in steady phase (day 3).

Proportion of *E. coli* resistant to fosfomycin, cephalosporins or meropenem during treatment.

Proportion of rectal colonization by Gram-negative rods producing ESBL or carbapenemases

4.2 Design

Open-label, randomized, multicenter, controlled, phase 3 clinical trial to compare the efficacy of the administration of intravenous fosfomycin 4 g / 6 hours administered in 1 hour, compared to intravenous meropenem 1g / 8 hours administered in 1 hour or intravenous ceftriaxone 1g / 24h administered in 2-4 minutes in the treatment of multiresistant E. coli bacteremic urinary infection.

4.3 Randomization

Patients will be detected from the daily review of blood culture results. In patients with isolation of multidrug-resistant *E. coli* in blood culture, the inclusion and exclusion criteria of the study will be verified and informed consent will be offered. If signed by the patient, randomization will be carried out, which will be centralized online, in a 1: 1 ratio (fosfomycin vs. comparator), and stratified by empirical treatment received (adequate or not adequate); in case the strain is resistant to quinolones but sensitive to cephalosporins, the patient will be randomized to fosfomycin or ceftriaxone; if it is resistant to cephalosporins, it will be randomized to fosfomycin or meropenem.

The system for randomization will be available online in the CRD designed for this purpose, the randomization list will be kept by the Clinical Trials Unit. Once you have entered that you meet all the inclusion criteria and none of the exclusion criteria, the demographic data and the previous empirical treatment, the researcher will obtain the assigned treatment and the patient code, which will be composed of a numerical code that identifies the center and patient.

4.4 Masking

There are no masking techniques, as it is an open study. Therefore, no procedure for breaking the blind is applicable either.

4.5 Study drugs

Experimental drug: fosfomycin disodium

Dose: 4 g / 6 hours
Pharmaceutical Form: powder for solution for injection 4g (Fosfomycin Level)
Therapeutic group: J01J3

Control drug 1: meropenem (anhidre/trihidrate)
Dose: 1 g / 8 hours
Pharmaceutical Form: powder for solution for injection and for infusion
Therapeutic group: J01D5

Control drug 2: ceftriaxone sodium
Dose: 1 g / 24 hours
Pharmaceutical Form: powder for solution for injection and for infusion
Therapeutic group: J01DD04

4.6 Follow-up

The follow-up of the patients will be carried out in the visits defined in this protocol and will continue until day 60 from the beginning. The visit schedule is specified below.

4.7 Criteria for termination or interruption of the Study

The premature interruption of the clinical trial may occur due to a decision of the regulatory

5.- SELECTION CRITERIA

Adult patients (18 years or older) hospitalized with urinary origin bacteremia due to multidrug-resistant E. coli sensitive to fosfomycin and meropenem or ceftriaxone. Candidates will be patients with multiresistant E. coli isolate in blood culture.

5.1 Inclusion criteria

Adult patients (18 years or older) hospitalized with multiresistant E. coli bacteremia of urinary origin, sensitive to fosfomycin and meropenem or ceftriaxone (see definitions below).

Isolation in blood culture of this microorganism with clinical data of sepsis with no other obvious or probable origin of the bacteremia other than the urinary one. Urinary origin requires at least one clinical criterion PLUS an analytical one among the following:

Clinical criteria:

- Presence of any lower urinary tract symptoms (dysuria, frequency, urgency, pain, suprapubic, tenesmus).
- Pain in the lumbar region.
- Positive renal fist-percussion.
- In the absence of the above, and given that bacteremia of urinary origin can occur in the absence of focal symptoms in some patients and that an analytical criterion is also required, bacteremia that occurs in the absence of symptoms and signs will be considered as urinary origin. from another source of infection. This is more common in catheterized patients and patients older than 70 years, but can occur at any age and circumstance.
- In patients older than 70 years, cognitive impairment in the absence of another cause can be considered a symptom of urinary infection.

Urinary analytical criteria

- Presence of pyuria (≥10 leukocytes / mm3 or positive leukocyte esterase in urine strip)
- Positive urine culture for the same pathogen isolated in blood according to standard criteria

Estimated need for intravenous treatment for at least 5 days.

Potentially fertile patients should have a negative pregnancy test. Patients who have given their written informed consent.

5.2 Criterios de exclusión

Polymicrobial bacteremia.

Undrained kidney abscess or unresolved urinary tract obstruction.

Hematogenous urinary tract infection or by contiguity.

Other concomitant infection.

Polycystic kidney disease.

Kidney transplant.

Known allergy or hypersensitivity to fosfomycin or meropenem or ceftriaxone.

Terminal situation, or estimated life expectancy of less than 90 days, or in purely palliative treatment for their underlying illness

Patients with a previous clinical diagnosis of chronic heart failure (CHF) of any functional grade of the NYHA (I to IV), liver cirrhosis, or renal failure requiring substitute treatments.

Patients in whom any of the following diagnoses (even in the absence of clinical manifestations of CHF) has been documented by a transthoracic echocardiography performed in the previous 6 months: any significant moderate or severe valvular disease, FEVi <35% or left ventricular hypertrophy with impaired relaxation.

ECG findings performed at baseline visit if:
- Presence of any rhythm disorder (atrial fibrillation or flutter) that had not been previously documented or that is not under specific treatment at the time of the baseline visit (patients with a previous diagnosis of atrial fibrillation or flutter are excluded from this last condition who have presented spontaneous control of the ventricular response up to that point without the need for cardio-braking treatment)
- Presence of findings compatible with left ventricular enlargement (according to the Sololow-Lyon criteria or other similar ones)
- Presence of corrected QT lengthening (QTc) according to heart rate (according to Bazet's formula).

Pregnant or lactating women.

Septic shock at the time of randomization.

Active empirical treatment> 72 hours after extraction of the initial blood culture.

Delay in inclusion> 24 hours after identification of multidrug-resistant E. coli in blood culture.

Patients who are participating in another clinical trial with active treatment.

5.3 Withdrawal criteria

In accordance with the Declaration of Helsinki, patients have the right to withdraw from the study at any time and for any reason, being able to express it personally or through their representative.

5.3.1 Efficacy criteria

Clinical failure. Clinical failure will be considered when any of the following circumstances occurs after 48 hours of treatment:

- Worsening of symptoms related to urinary tract infection.

- No disappearance of the sepsis situation (if it was present at the beginning).
- Appearance of new symptoms of urinary infection.
- Need to suspend the antibiotic or add another due to lack of efficacy based on the above criteria.

Microbiological withdrawal criteria: positive blood culture after 5 days of treatment.

5.3.2 Safety criteria

Any adverse event that at the discretion of the clinician forces the withdrawal of the study antibiotic.

When, for any reason, the treatment is no longer safe for the patient. Or for any other reason that could endanger the life of the patient or have serious consequences for the patient.

Patient with a firm diagnosis of prostatitis that, in the opinion of the investigator, requires more than 14 days of treatment.

Diagnosis of kidney abscess after inclusion in study

5.3.3 For breach or violation of the rules contained in the protocol

When the patient stops complying with the rules of the trial, they may be withdrawn at the discretion of the responsible investigator or due to loss of follow-up.

If there is a need to introduce an antibiotic with activity against Gram negative bacilli, other than those studied, for any reason before the end of the trial treatment.

5.3.4 Follow-up of patients with early withdrawal

If a patient is withdrawn from the trial prematurely, the investigator will provide the main reason for the suspension and, as indicated by the GCP guidelines, the procedures will be followed according to the usual treatment protocols for their pathology at the discretion of the responsible clinician.

5.4 Additional definitions

Multi-resistant *E. coli*: *E. coli* showng in vitro resistance to at least one drug from at least three families of antibiotics, including: aminoglycosides, penicillins, penicillins with beta-lactamase inhibitors, antipseudomonal with beta-lactamase inhibitors, 1st and 2nd generation cephalosporins, 3rd and 4th generation cephalosporins, cephamycins, monobactams, fluoroquinolones, inhibitors of the folate pathway, tetracyclines or phenicols [24].

Early clinical cure: Early clinical cure is defined analogously to that explained above for clinical cure at the cure visit, but assessed after 5 full days of treatment (visit 3).

<u>Clinical failure.</u> Any of the following:

- No disappearance or return to the baseline situation of all clinical criteria present at the beginning.

- Failure of all the symptoms and signs of desepsis.
- Appearance of new symptoms.
- Need to suspend the antibiotic or add another due to lack of efficacy based on the previous criteria.

- Death.

Microbiological failure: positive blood culture on day 5 and / or positive urine culture on day 5 or in the test of cure.

6.- PARTICIPANTS TREATMENT

Experimental treatment:

Fosfomycin disodium in powder form for solution for injection. It will be administered intravenously, in a 1-hour drip at a rate of 4g every 6 hours.

Preparation and infusion: Dissolve the content of the 4 g vial with 20 mL of water for injection. Pass through a container with 230 Ml of 5% glucose and put in a regulated gravity drip system (“Dosiflow®” type) lasting 1 hour at a rate of 250ml / h (total volume of 250 mL).

NOTE: Dissolving fosfomycin produces an exothermic reaction, with consequent release of heat, causing the vial to warm slightly. The intravenous fosfomycin solution in glucose serum is stable for 24 hours at room temperature (25 ± 2ºC).

Control treatment 1:

Meropenem in powder form for solution for injection and for infusion. It will be administered intravenously, in a drip lasting 15-30 minutes at a rate of 1g every 8 hours.

Preparation and infusion: dissolve the content of the 1 g meropenem vial with 20 mL of water for injection. Add the reconstituted vial (20 mL) to a 100 mL bag of 0.9% sodium chloride. The administration will be carried out by means of a regulated gravity drip system (type “Dosiflow®”) lasting 15-30 minutes at a constant rate to infuse said volume in the specified time (total volume of 120 mL).

NOTE: The stability of the reconstituted vial is 3 hours at room temperature (25 ± 2ºC). The stability of the infusion solution is 6 hours at room temperature (25 ± 2ºC) and 24 hours between 2-8ºC

Control treatment 2:

Ceftriaxone in powder form for intravenous injectable solution. It will be administered intravenously, at a dose of 1 g per day, in 2-4 minutes.

Preparation and infusion: Reconstitute each 1g vial in 10ml of water for infusion or 0.9% sodium chloride. Avoid using solutions containing calcium (eg Ringer or Hartam).

Sequential treatment

From the 5th day of treatment with the study medication, if all the necessary conditions are met, sequential treatment (oral or parenteral) is allowed until a minimum of 10-14 days of treatment are completed. The necessary conditions to be able to propose an oral or intramuscular treatment are: Clinical improvement, hemodynamic stability, tolerance to oral intake if an oral drug is to be administered, and there is an appropriate treatment option according to the antibiogram.

Experimental group: oral Fosfomycin trometamol 3 g every 48 hours.

Control group: the following drugs and in this order, based on the sensitivity of the strain:
In the case of a sensitive cephalosporin strain: Cefuroxime axetil, 250 mg every 12 hours
In case of cephalosporin resistant strain:

- Ciprofloxacin 500 mg / 12hv.o.
- Amoxicillin / clavulanate 500 mg / 8hv.o.
- Trimethoprim-sulfamethoxazole 160 / 800mg / 12h.v.o.
- Ertapenem 1g / 24hparenteral
- Amikacin 15 mg / Kg / day parenteral

6.1 Dosing adjustement in case of renal insufficiency

The dose of the different antibiotics administered should be adjusted based on creatinine clearance calculated through the Cockoft-Gault formula.

Fosfomycin disodium: The dosage adjustment in the group of patients treated with fosfomycin is carried out by maintaining a dose of 4g per administration, but increasing the interval between them as shown in the following table:

Clearance creatinine (ml/min)	Dose	Frequency
**40-20**	4gr	12h
**20-10**	4gr	24h
**≤ 10**	4gr	48h

Meropenem

Creatinin clearance (ml/min)	Dose	Frequency
**26-50**	1gr	12h

**10-25**	500mg	12h
**<10**	500mg	24h

Ceftriaxone

No adjustment is needed in the context of this study

Ciprofloxacin

Creatinine clearance (ml/min)	Doe	Frequency
**>60**	500mg	12h
**30-60**	250-500mg	12h
**<30**	250-500mg	24h

Amoxicillin-clavulanate

Creatinin clearance (ml/min)	Dose	Frequency
**10-30**	500/125mg	12h
**<10**	500/125mg	24h

Trimethoprim-sulfamethoxazole

Creatinina clearance (ml/min)	Dose	Frequency
**>30**	160/800mg	12h
**15-30**	80/400mg	12h
**<15**	Not recommended	Not recommended

Ertapemen

Creatinine clearaance (ml/min)	Dose	Frequency

**>30**	1g	24h
**< 30**	Not recommended	Not recommended

<u>Amikacin</u>

Creatinina clearance (ml/min)	Dose	Frequency
**>50**	15mg/kg	24h
**10-50**	10mg/kg	24h
**< 10**	10mg/kg	48h

<u>Fosfomycin trometamol</u>

In patients with mild to moderate renal impairment, it is not necessary to modify the dose within the recommended dosage range since its therapeutic concentration in urine remains unchanged.

6.2 Concomitant medication

The concomitant use of systemic antibiotics with activity against Gram negative bacilli will not be allowed. The administration of this type of drugs in the phase in which antibiotic treatment lasts will be the criterion for withdrawal from the study. Its administration in the post-treatment follow-up phase will be taken into account in the statistical analysis.

6.3 Rescue medication

The use of rescue medication is not planned. If a patient is withdrawn due to lack of efficacy or some similar condition, they will be treated according to the guidelines clinical practice and routine clinical practice for these cases.

6.4 Schedule of visits

Visit	**1**	**2**	**3**	**4**	**5**	**6**	**7**
Day	**1**	**3**	**5-7**	**12±2 (end of therapy)**	**5-7 after end of treatment (test of cure)**	**60±10**	**Unscheduled visit(s)**
Informed consent	x						
Inclusion/exclusión criteria	x						
Pregancy test	x						
Randomization	x						
Clinical history, anamnesis	x	x	x	x	x	X	x
Physical examination	x	x	x	x	x	(x)	x
Blood count/chemistry[1]	x	x	x	x			x
Urine (elementary)	x		x		x		x
Urine culture	x		x		x		x
Blood culture	x	x	x[2]				x
Urinary tract ultrasound	X						x
Electrocardiogram	x						x
PK/PD samples[3]		x					x
Rectal swab[4]	x		x	x			
Urinary catheter change of present	x						
Medication dispensing	x	x	x	x			
Adverse events reporting		x	x	x	x	x	x
Concomitant medication checking		x	x	x	x	x	x

1. Full blood cell count; glucose, sodium, potassium, creatinine, bilirrubine, AST, ALT, CRP
2. Only if previous was positive
3. Only at Hospital Universitario Virgen Macarena
4. Only at Hospital Universitario Virgen Macarena, Hospital Bellvitge and Hospital Vall d´Hebrón

6.5 Procedures per visit

Day 1 is the day of recruitment and start of study treatment.

6.5.1 Visit1 (Day 1)

o Identification of cases through the microbiology laboratory of each center.

o Signature of the informed consent.

o Assessment of the inclusion / exclusion criteria.

o In the case of women of childbearing age, a pregnancy test will be requested.

o Anamnesis with collection of personal history: Charlson index, McCabe index, previous urological and renal pathology, previous urinary procedures (including catheterization) and symptoms of urinary infection.

o Demographic data.

o Antibiotherapy received in the 7 days prior to inclusion and especially those received to treat the current episode of urinary infection, assessing its in vitro activity against the isolated strain.

o Physical examination with collection of weight, height, blood pressure, heart rate, respiratory rate, temperature, renal fist-percussion, abdominal palpation, presence of edema and status.

o Score in the Pitt score (day of extraction of the initial blood culture that allows the identification of the case).

o Classification on the sepsis scale (no sepsis, sepsis, severe sepsis, septic shock, at the time of case identification).

o Form of acquisition of bacteremia (nosocomial, community or associated with health care).

o Hematology: with total leukocyte, neutrophil and platelet count. Coagulation study (if not done within 72 hours).

o Blood chemistry: sodium, creatinine, urea, GPT, GOT, CRP and total bilirubin (and direct bilirubin if totally altered)

o Urinalysis: urine leukocyte count (or determination of leukocyte esterase using test strips) and presence of nitrites.

o Urine culture: it should be done before the start of the study treatment.

o Blood culture. It should be done prior to the infusion of the study medication. o Rectal smear. This test will be performed only on hospital patients Virgen Macarena, Bellvitge and Valld´Hebrón.

o Renal ultrasound. If it had not been performed in the 72 hours prior to inclusion, a renal ultrasound should be requested. It can be postponed for up to 5 days after inclusion.

o Electrocardiogram: baseline 12-lead ECG should be performed

o Change of urinary catheter (urethral or nephrostomy catheter). In those patients who had a urinary catheter at the beginning of the current condition, the change (or withdrawal if appropriate) of the urinary catheter should be carried out if said change (or withdrawal) has not been carried out in the 72 hours prior to inclusion.

o Control of dispensing / administration of medication.

o Rectal swab: only in designated sites

6.5.2 Visit 2 (Day 2)

o Anamnesis: as above

o Physical examination: as above

o Hematology, blood chemistry: as above

o Blood culture.

o Pharmacokinetic study (only in Hosptital Universitario Virgen Macarena

o Control of the dispensing and administration of the medication.

o Review of concomitant medication.

o Assessment of adverse events.

6.5.3 Visit 3 (Day 5-7)

o Anamnesis: as above

o Physical examination: as above

o Hematology, blood chemistry: as above

o Blood culture (only if previous is positive), urine culture.

o Urine (elementary): leucocytes or esterase, nitrites

o Control of the dispensing and administration of the medication.

o Review of concomitant medication.

o Assessment of adverse events.

o Evaluation for sequential terapy

o Rectal swab: only in designated sites

6.5.4 Visit 4. End of therapy (Day12±2)

o Anamnesis: as above

o Physical examination: as above

o Hematology, blood chemistry: as above

o Blood culture (only if previous is positive), urine culture.

o Urine (elementary): leucocytes or esterase, nitrites

o Control of the dispensing and administration of the medication.

o Review of concomitant medication.

o Assessment of adverse events.

o Rectal swab: only in designated sites

6.5.5 Visit 5. Test of cure (5-7 days after end of treatment)

o Anamnesis: as above

o Physical examination: as above

o Hematology, blood chemistry: as above

o Blood culture (only if previous is positive), urine culture.

o Urine (elementary): leucocytes or esterase, nitrites

o Control of the dispensing and administration of the medication.

o Review of concomitant medication.

o Assessment of adverse events.

o Rectal swab: only in designated sites

o Information about recurrences. Provide phone contact data.

6.5.6 Visit 6 (day 60±10)

This visit can be done in person or by telephone, with the patient sending the urine sample for analysis and urine culture through a peripheral center according to the usual clinical sample delivery circuit in each center. A similar procedure is allowed for rectal smear.

o Anamnesis: as above

o Review of concomitant medication.

o Assessment of adverse events.

6.5.7 Unscheduled visit(s)

If a patient develops symptoms consistent with urinary tract infection / bacteremia between visit 5 (cure test) and visit 6 (late post-treatment visit on day 60), an unscheduled visit should be performed within 48 hours. To do this, the patient must have a telephone number to contact the research team in case of recurrence of symptoms. In the unscheduled visit the following procedures will be performed:

o Anamnesis: as above

o Physical examination: as above

o Hematology, blood chemistry: as above

o Blood culture, urine culture.

o Urine (elementary): leucocytes or esterase, nitrites

o Control of the dispensing and administration of the medication.

o Review of concomitant medication.

o Assessment of adverse events.

o Rectal swab: only in designated sites

o Information about recurrences. Provide phone contact data.

7. MICROBIOLOGICAL STUDIES

E. coli isolates from blood and urine cultures

These are fundamental studies for the detection of study patients and the measure of efficacy. They will be extracted locally following the clinical circuits of each center. Identification and sensitivity testing (EUCAST recommendations) should be carried out in local laboratories. In addition, the microbiological isolates must be sent to the coordinating center for a centralized study (study of the sensitivity to fosfomycin, fluoroquinolones, cephalosporins and meropenem by reference techniques and characterization of ESBL or AmpC by PCR and sequencing).

To study the mechanisms of resistance to fosfomycin in resistant strains that are isolated in the course of treatment with this antimicrobial, mechanisms of decreased penetration of fosfomycin into bacteria, modification of the target, and inactivation of the antibiotic would be studied. The characterization of the genes involved would be done using PCR and sequencing techniques.

Rectal colonization studies

In 3 centers participating in the trial (Hospital Virgen Macarena, Bellvitge and Vall d'Hebrón), a study of the intestinal flora will be carried out by obtaining rectal smears at visits 1m 3 and 4, with the aim of determine the presence of ESBL- or carbapenemase-producing Enterobacteriaceae (n ≈ 60 patients).

The swabs will be inserted into the tubes containing the transport medium and labelled. Amies, Stuart, or Cary-Blair transport medium tube swab will be used. The samples will be transported to the local microbiology laboratory immediately.

The rectal swabs will be inoculated in McConkey medium containing cefotaxime (2 mg/L) or Chrom ID ESBL (BioMerieux, France). All isolates obtained will be identified using routine microbiology methods, frozen at -80ºC and shipped to the reference laboratory, where species identification will be confirmed using MALDI Biotyper (Bruker Daltonics, Billerica, Massachussetts, USA) according to the manufacturer instructions. ESBL-producers will be confirmed using the double-disc sinergy test (Liofilchelm). Susceptibility testing will be performed by microdilution.

8. PHARMACOKINETIC STIDUES

At Hospital Universitario Virgen Macarena, blood levels of fosfomycin will be measured at day 3 in 20 patients (see specific protocol).

9.- SAFETY ASSESSMENT

9.1 Safety evaluations

Physical examinations including viral signs
Laboratory tests
Urinary tract ultrasound
Electrocardiogram

9.2 Adverse events

Diarrhea: those subjects who experience diarrhea (≥3 stools per day of decreased consistency) during the study will be asked to detect *Clostridium difficile* toxin in stool. If this is positive, it will be registered as AA. Although it will not force to suspend the treatment of the study, unless the IP considers it so

Seizures

Hepatic toxicity.

Hemolytic anemia and other haematological alterations.

Edema, volume overload, hypernatremia. Especially in the fosfomycin arm (each 4 gram vial of fosfomycin disodium contains 1320 mg of sodium).

9.3 Definitions

Adverse event (AA): It is any incidence that is harmful to health in a patient or subject of a clinical trial treated with a drug, although it does not necessarily have a causal relationship with treatment. An adverse event can therefore be any unfavorable and unintended sign (including an abnormal laboratory finding), symptom or disease temporarily associated with the use of an investigational medicinal product, whether or not it is related to the investigational medicinal product.

Adverse Reaction (AR): An adverse reaction is considered any harmful and unintended reaction to an investigational drug, regardless of the dose used.

Imputability Criteria: The promoter will classify AA, based on their causal relationship with the drug, as:

- Definitive: there is a reasonable time sequence between the administration of the drug and the appearance of AA. This event coincides with the ARs described for the drug, improves with its withdrawal, reappears after its re-administration, and cannot be explained by alternative causes.

- Probable: there is a reasonable time sequence between the administration of the drug and the appearance of AA. This event coincides with the ARs described for the drug, improves after discontinuation of treatment, and cannot be explained by other alternatives

- Possible: there is a reasonable time sequence between the administration of the drug and the appearance of AA. This event coincides with the ARs described for the drug but can be explained by alternative causes.

- Conditional or Unlikely: there is a reasonable time sequence between the administration of the drug and the appearance of AA. This event does not coincide with the ARs described for the drug and can be explained by alternative causes.

- Unrelated: there is no reasonable time sequence between the administration of the drug and the appearance of AA. This event does not coincide with the ARs described for the drug and can be explained by alternative causes.

The determination of the possible relationship with the study treatment is the responsibility of the principal investigator of the research center or the person designated by it.

Severity: Any adverse event or adverse reaction is considered serious if, at any dose:
- Cause the death of the patient
- Threatens the patient's life1
- Require hospitalization or prolongation of the patient's hospitalization
- Causes permanent or significant disability or disability
- Resulting in a congenital anomaly or malformation

9.4 Notification and collection of serious adverse events

In the event of a serious adverse event (AAG) that must be notified to the Promoter using the form designed for this purpose, a member of the investigation team will complete and sign the AAG notification form that will be sent by e-mail (from the staff responsible for monitoring), immediately and always within 24 hours after having knowledge of the event:

Notificación y recogida de acontecimientos adversos graves
Unidad de Investigación Clínica y Ensayos Clínicos
Hospital Universitario Virgen del Rocío
Avda. Manuel Siurot S/N
41013. Sevilla
Email: pv_forest@scren.es

The promoter will notify the Spanish Agency for Medicines and Health Products of all suspected serious and unexpected adverse reactions associated with investigational drugs. The maximum notification period will be 15 calendar days from the moment the promoter has become aware of the suspected adverse reaction.

When the suspicion of a serious and unexpected adverse reaction has caused the death of the subject, or endangered his life, the promoter will inform the Spanish Agency of Medicines and Health Products within a maximum period of seven calendar days from the moment the promoter is aware of the case. Said information must be completed, if possible, in the following eight days.

9.5 Notification to researchers

The sponsor will communicate to the investigators any information that may affect the safety of the trial subjects as soon as possible.

10.- SAMPLE SIZE AND STATISTICAL ANALYSES

10.1 Sample size

To calculate the sample size, for a power> 80% with an alpha error, <5%, taking into account an estimated proportion of clinical cure of 90% in the control group and 85% in the experimental group, assigning in proportion 1: 1, for a loss level of 5% and accepting a non-inferiority limit of 7%, 198 patients will be needed (99 in each group).

In the feasibility survey carried out in the selected centers, all of them presented more than 15 episodes of bacteremic urinary tract infection by E. coli ESBL in 2012 that were included, and they agreed to include at least 10 patients in the study period, competitively with which the achievement of the sample size is guaranteed. Despite this, the inclusion recorded in the first months of

10.2 Statistical analyses

The absolute difference in the percentages of clinical and microbiological cure at the cure visit (5-7 days after completion of treatment) between the patients in both arms and their 95% confidence interval will be calculated.

A multivariate analysis will be performed using logistic regression for the main outcome variable to ensure the independence of the treatment effect. In said multivariate analysis, special consideration will be given to the study that the center of origin of the case may have.

The absolute difference in the percentages of early clinical cure and early microbiological cure (5th days of treatment) between both arms and their 95% confidence interval will be calculated.

In a similar way, we will proceed with other variables studied, such as mortality, hospital stay, the proportion of adverse effects, the proportion of recurrences and changes in rectal colonization by multidrug-resistant organisms.

A description will be made of the plasma concentrations of fosfomycin and the area under the curve. A description will also be made of the frequency of appearance of fosfomycin resistant strains in clinical and epidemiological samples (rectal smears).

10.3 Interim analysis

An interim analysis is planned when 50% of the cases have been recruited. In this analysis, an independent committee of 3 experts will also carry out a safety assessment.

10.4 Study populations

Intent-to-treat (PITT) population: all randomized patients.

Modified intention-to-treat population (mITT): randomized patients who have received at least one intravenous dose of antibiotics.

Clinically evaluable population (PCE): patients who have completed 5 days of intravenous treatment (or who have died before, after having received at least one dose of intravenous antibiotic) and a total duration of at least 10 days, with at least 75% of taking oral antibiotics if sequential treatment was performed.

Clinically and microbiologically evaluable population (PCME): refers to the clinically evaluable population in which the microbiological tests (blood culture and urine culture, if applicable) have been performed in the visits scheduled during the follow-up until the evaluation.

10.5 Independent evaluation committee

To avoid as much as possible the bias that the open nature of the trial could generate, the evaluation of the results will be carried out by an independent committee blinded regarding the allocation of treatment. This committee will be made up of 3 expert researchers belonging to the Spanish Network for Research in Infectious Pathology (REIPI) and will take its conclusions by consensus.

11.- ETHIC ASPECTS

The trial will be carried out in accordance with the principles that emanate from the Declaration of Helsinki, and according to current legal regulations (Royal Decree 1090/2015), and will not begin until the approval of the reference CEIC, the conformity of the Directors of the Institutions, and the authorization of the Spanish Agency for Medicines and Health Products.

The investigator must meet all the requirements of the protocol. If a situation arises in which a temporary deviation from the protocol is required, the investigator or other physician responsible for the patient should contact the monitor as soon as possible in order to comment on the situation and agree on an appropriate course of action. The investigator will document the deviation from protocol and the circumstances that required it.

11.1 Consentimiento informado

The patient must give their consent before being admitted to the clinical study. The doctor will have to explain the nature, purposes and possible consequences of the clinical trial, in a way that is understandable to the patient. The information provided by the doctor must also be recorded. In obtaining and documenting it, the researchers will comply with the pertinent legislation (article 4 of RD 1090/2015), the rules of good clinical practice and the ethical principles that have their origin in the Declaration of Helsinki.

The subject of the study will give his consent, signing the corresponding model. The researcher will receive an adequate number of informed consent templates through the Sponsor. To this end, each model must bear the signature of the researcher and the patient.

The investigator will not initiate any investigation for the trial until the consent of the patient has been obtained.

11.2 Data protection

The treatment, communication and transfer of personal data of all participating subjects will comply with the provisions of REGULATION (EU) 2016/679 OF THE EUROPEAN PARLIAMENT AND OF THE COUNCIL of April 27, 2016 regarding the protection of natural persons with regard to the processing of personal data and the free circulation of these data and by which Directive 95/46 / EC (General Data Protection Regulation) is repealed. In accordance with the provisions of the aforementioned legislation, the patient may exercise the rights of access, modification, opposition and cancellation of data, for which he must contact his study doctor.

The anonymity of the subjects participating in the study will be maintained at all times. Thus, the data collected for the study will be identified by a code (see point 6.3) and only the researcher and collaborators will be able to relate said data to the patient and their medical history. Therefore, the identity of the patient will not be revealed to any person except for exceptions: personnel authorized by the promoter, when required, to verify the study data and procedures, but always maintaining their confidentiality in accordance with current legislation; in case of medical emergency or legal requirement (health

authorities: Spanish Agency for Medicines and Health Products and Local Committee for Clinical Trials).

Data from this study will be used only for the specific purposes of this study.

11.3 Monitoring and auditing

The study will be monitored through local visits, telephone calls and periodic inspection of the CRDs with enough frequency to verify the following:

Rate of inclusion of patients, compliance with the rules of the protocol procedures, integrity and accuracy of the data entered in the notebooks, verification against the original documents and appearance of adverse events.

The monitoring visits will be made by the studio monitors. It is understood that these monitors will be able to access the clinical histories of the patients after the investigator requests it. The researcher will dedicate sufficient time to these visits and will facilitate access to all the documentation to the authorized persons.

The study may be audited by an independent body. In the same way, members of the CEIC will be able to follow up on it.

12.- FUDING AND INSURANCE

3.1 Financing

The project has received funding through a public call for Strategic Action in Health from the Instituto de Salud Carlos III 2013. Due to its eminently non-commercial nature, the Hospital provides the human material and material resources necessary to carry out the study, including experimental drugs and control

13.2 Insurance

The promoter has contracted a civil liability insurance policy in accordance with the requirements specified in article 9 of RD1090 / 2015.

13.- PUBLICATION POLICY

These will comply with the provisions of Royal Decree 1090/2015 of December 4, which regulates Clinical Trials with medicines, the Ethics Committees for Research with medicines and the Spanish Registry of Clinical Studies, article 42.

REFERENCES

1. Rodríguez-Baño J, Pascual A. Clinical significance of ESBL. Expert Rev Anti Infect Therapy 2008;6:671-683.

2. Rottier WC et al. Effects of confounders and intermediates on the association of bacteraemia caused by extended spectrum beta-lactamase-producing *Enterobacteriaceae*. J Antimicrob Chemother 2012;67:1311-1320.

3. Rodríguez-Baño J et al. Community-onset bacteraemia due to extended-spectrum beta-lactamase-producing *Escherichia coli*: risk factors and prognosis. Clinical infectious diseases 2010;50:40-48.

4. Tzouvelekis LS et al. Carbapenemases in *Klebsiella pneumoniae* and other *Enterobacteriaceae*: an evolving crisis of global dimensions. Clin Microbiol Rev 2012;25:682-706.

5. Vidal L, et al. Efficacy and safety of aminoglycoside monotherapy: systematic review and meta-analysis of randomized controlled trials. J Antimicrob Chemother 2007;60:247-257.

6. Docobo-Pérez F, et al. Inoculum effect on the efficacies of amoxicillin-clavulanate, piperacillin-tazobactam, and imipenem against extended-spectrum β-lactamase (ESBL)-producing and non-ESBL-producing *Escherichia coli* in an experimental murine sepsis model. Antimicrob Agents Chemother 2013; 57:2109-2113.

7. López-Cerero L, et al. Comparative assessment of inoculum effects on the antimicrobial activity of amoxicillin clavulanate and piperacillin-tazobactam. Clin Microbiol Infect 2010;16:132-136.

8. Rodríguez-Baño J et al. B-lactam/B-lactam inhibitor combinations for the treatment of bacteremia due to extended-spectrum B-lactamase-producing *Escherichia coli*. Clin Infect Dis2012;54:167-174.

9. Retamar P, et al. Impact of the MIC of piperacillin-tazobactam on the outcome of patients with bacteremia due to extended-spectrum-B-lactamase-producing *Escherichia coli*. Antimicrob Agents Chemother 2013;57:3402-4304.

10. Falagas ME et al. Antimicrobial susceptibility of multidrug-resistant (MDR) and extensively drug-resistant (XDR) *Enterobacteriaceae* isolates to fosfomycin. Int J Antimicrob Agents 2010; 35:240-243.

11. De Cueto M et a. In vitro activity of fosfomycin against extended-spectrum-beta-lactamase-producing Escherichia coli and *Klebsiella pneumoniae*. Antimicrob Agents Chemother2006;50:368-70.

12. Oteo J et al. Parallel increase in community use of fosfomycin and resistance to fosfomycin in extended-spectrum beta-lactamase (ESBL)-producing *Escherichia coli*. J Antimicrob Chemother 2010;65:2459-2463.

13. Karageorgopoulos DE et al. Fosfomycin: evaluation of the published evidence on the emergence of antimicrobial resistance in Gram-negative pathogens. J Antimicrob Chemother 2010;67:255-268.

14. Falagas ME et al. Fosfomycin for the treatment of multidrug-resistant, including extended-spectrum betalactamase producing, Enterobacteriaceae infections: a systematic review. Lancet Infect Dis 2010; 10: 43-50.

15. Pulcini et al. Forgotten antibiotics: an inventory in Europe, the United States,Clin Infect Dis 2012; 54:268-274.

16. Rex et al. A comprehensive regulatory framework to address the unmet need for new antibacterial treatments.Lancet Infect Dis 2013;13:269-275.

17. Infectious Diseases Society of America. White Paper: recommendations on the conduct of superiority and organism-specific clinical trials of antibacterial agents...Clin Infect Dis 2012; 55:1031-1046.

18. Retamar P et al. Impact of inadequate empirical therapy on the mortality of patients with bloodstream infections: a propensity score-based analysis. Antimicrob Agents Chemother 2012; 56:472-478.

19. Roussos N, et al. Clinical significance of the pharmacokinetic and pharmacodynamics characteristics of fosfomycin for the treatment of patients with systemic infections.Int J Antimicrob Agents 2009;34:506-515.

20. Matzi V et al. Extracellular concentrations of fosfomycin in lung tissue of septic patients. J Antimicrob Chemother2010;65:995-998.

21. Rodríguez-Baño J et al. Community infections caused by extended-spectrum-lactamase producing-*Escherichia coli*. Arch Intern Med 2008;168:1897-1902.

22. Gardiner BJ, et al. Is fosfomycin a potential treatment alternative for multidrug-resistant Gram-negative prostatitis? Clin Infect Dis. 2014; 58:e101-5.

23. FDA. Complicated urinary tract infections: developing drugs for treatment. Draft guidance. February2012.

24. Magiorakos AP, et al. Multidrug-resistant, extensively drug-resistant and pandrug-resistant bacteria: an international expert proposal for interim standard definitions for acquired resistance. Clin Microbiol Infect. 2012Mar;18(3):268-81.

PARTICIPATING SITES

Hospital Universitario Virgen Macarena (Sevilla). IP: Jesús Rodríguez Baño

Hospital General Universitario de Alicante (Alicante). IP: Esperanza Merino.

Hospital de Cruces (Baracaldo). IP: Elena Bereciartua Bastarrica.

Hospital Parc Salud Mar (Barcelona). IP: María Luisa Sorlí Redó.

Hospital de la Santa Creu i San Pau (Barcelona). IP: Virginia Pomar.

Hospital Vall d´Hebron (Barcelona). IP: Carlos Pigrau Serrallach.

Hospital Unversitario de Bellvitge. IP: Evelyn Shaw Perujo.

Hospital Universitario Reina Sofía (Córdoba). IP: Clara Natera Kindelan.

Hospital Universitario de Canarias (La Laguna). IP: María Lecuona Fernández.

Hospital Universitario de Gran Canaria Dr. Negrín (Gran Canaria). IP: Miguel Ángel Cárdenas Santana.

Hospital Arnau de Vilanova (Lérida). IP: Alfredo Jover Sáenz.

Hospital Universitário 12 de Octubre (Madrid). IP: Rafael San Juan Garrido

Hospital Ramón y Cajal (Madrid). IP: Vicente Pintado García.

Hospital Clínico Universitario Virgen de la Arrixaca (Murcia). IP: Alicia Hernández Torres.

Hospital Marqués de Valdecillas (Santander). IP : Jorge Calvo Montes

Hospital Universitario de Terrassa (Tarrasa). IP: Esther Calbo.

Hospital Marina Baixa (Villajoyosa, Alicante). IP: Concepción Amador Prous.

Hospital Regional Universitario de Málaga (Carlos Haya). IP: Jose María Reguero Iglesias

Hospital Universitario Virgen del Rocío. IP: José Molina-Gil Bermejo
