# Supplementary material for: Effectiveness of Fosfomycin for the Treatment of Multidrug-Resistant Escherichia coli Bacteremic Urinary Tract Infections: A Randomized Clinical Trial
Source: JAMA Netw Open. 2022 Jan 13;5(1):e2137277. doi: 10.1001/jamanetworkopen.2021.37277 (PMC8759008; doi:10.1001/jamanetworkopen.2021.37277)
Supplement: Supplement 2. — eTable 1. Baseline Characteristics of Patients in Modified Intention-to-Treat Population With Ceftriaxone-Susceptible Isolates by Fosfomycin vs Ceftriaxone eTable 2. Baseline Characteristics of Patients in Modified Intention-to-Treat Population With Ceftriaxone-Resistant Isolates by Fosfomycin vs Meropenem eTable 3. Extended-Spectrum β-Lactamases Produced by Ceftriaxone-Resistant Isolates at Baseline eTable 4. Patients Who Did Not Reach Clinical and Microbiological Cure at Test of Cure for Reasons Other Than Clinical or Microbiological Failure eTable 5. Analyses of Clinical or Microbiological Failure Rates at Test of Cure in Subgroups of Modified Intention-to-Treat Population eTable 6. Clinical and Microbiological Cure and Relapse Rates Among Patients Who Switched to Oral Drugs or Parenteral Ertapenem eTable 7. Multivariate Analysis of Variables Associated With Clinical and Microbiological Cure in Modified Intention-to-Treat Population eTable 8. Adverse Events Reported eTable 9. Description of Serious Adverse Events eTable 10. Ceftriaxone-Resistant Bacteria Isolated After Treatment [file jamanetwopen-e2137277-s002.pdf]

Supplemental Online Content

Sojo-Dorado J, López-Hernández I, Rosso-Fernandez C, et al; REIPI-GEIRAS-FOREST group. Effectiveness of fosfomycin for the treatment of multidrug-resistant *Escherichia coli* bacteremic urinary tract infections: a randomized clinical trial. *JAMA Netw Open*. 2022;5(1):e2137277. doi:10.1001/jamanetworkopen.2021.37277

eTable 1. Baseline Characteristics of Patients in Modified Intention-to-Treat Population With Ceftriaxone-Susceptible Isolates by Fosfomycin vs Ceftriaxone

eTable 2. Baseline Characteristics of Patients in Modified Intention-to-Treat Population With Ceftriaxone-Resistant Isolates by Fosfomycin vs Meropenem

eTable 3. Extended-Spectrum β-Lactamases Produced by Ceftriaxone-Resistant Isolates at Baseline

eTable 4. Patients Who Did Not Reach Clinical and Microbiological Cure at Test of Cure for Reasons Other Than Clinical or Microbiological Failure

eTable 5. Analyses of Clinical or Microbiological Failure Rates at Test of Cure in Subgroups of Modified Intention-to-Treat Population

eTable 6. Clinical and Microbiological Cure and Relapse Rates Among Patients Who Switched to Oral Drugs or Parenteral Ertapenem

eTable 7. Multivariate Analysis of Variables Associated With Clinical and Microbiological Cure in Modified Intention-to-Treat Population

eTable 8. Adverse Events Reported

eTable 9. Description of Serious Adverse Events

eTable 10. Ceftriaxone-Resistant Bacteria Isolated After Treatment

This supplemental material has been provided by the authors to give readers additional information about their work.

© 2022 Sojo-Dorado J et al. *JAMA Network Open.*

eTable 1. Baseline Characteristics of Patients in Modified Intention-to-Treat Population With Ceftriaxone-Susceptible Isolates by Fosfomycin vs Ceftriaxone[a]

Characteristic	Fosfomycin (n=39)	Ceftriaxone (n=31)
Age in years, median (interquartile range)	68 (60-78)	72 (60-81)
Female sex	19 (61.3)	16 (51.6)
Charlson index, median (interquartile range)	1 (0-2)	1 (0-2)
Charlson index ≥3	7 (22.6)	6 (19.4)
Congestive heart failure	4 (12.9)	0
Chronic pulmonary Disease	4 (12.9)	4 (12.9)
Diabetes mellitus	9 (29.0)	8 (25.8)
Chronic renal disease	2 (6.5)	5 (16.1)
Cancer	7 (22.6)	4 (12.9)
Full dependence for basic activities	3 (9.7)	0
Urinary catheter	8 (25.8)	9 (29.0)
Invasive procedure in the urinary tract in previous month	6 (19.4)	3 (9.7)
Immunosupressant drugs	3 (9.7)	1 (1.3)
Present infection		
Community-acquired infection	13 (41.9)	22 (71.0)
Healthcare-associated infection	15 (48.4)	6 (19.4)
Nosocomial infection	3 (9.7)	3 (9.7)
Low-urinary tract symptoms	17 (54.8)	21 (67.7)
Lumbar pain/tenderness	12 (38.7)	15 (48.4)
Severe sepsis at presentation	6 (19.4)	6 (19.4)
Pitt score, median (interquartile range)	1 (0-1)	1 (0-2)
Creatinine clearance ≤60 ml/min	7 (22.6)	9 (29.0)
Positive blood culture at recruitment day	3 (9.7)	2 (6.5)
Hydronephrosis in echography	3 (9.7)	4 (12.9)
Active treatment in ≤1 day since blood culture	26 (83.9)	25 (80.6)
Mean days until active treatment (SD)	1·3 (1.3)	1.2 (1.1)
Mean days until randomization (SD)	2·5 (0.7)	2·6 (0.8)
Removal/change of urinary catheter in ≤48h	10/11 (90·9)	6/8 (75.0)
Susceptibility of baseline *E. coli* (local laboratory)		
Amoxicillin	21 (67.7)	27 (87.1)
Amoxicillin-clavulanic acid	22 (71.0)	17 (54.8)
Piperacillin-tazobactam	29 (93.5)	26 (83.9)
Cefotaxime	31 (100)	31 (100)
Cefepime	31 (100)	31 (100)
Meropenem	31 (100)	31 (100)
Ciprofloxacin	7 (22.6)	5 (16.1)
Trimethoprim-sultamethoxazole	16 (51.6)	9 (29.0)
Amikacin	30 (96.8)	31 (100)
Fosfomycin	31 (100)	31 (100)
Baseline *E. coli* is ESBL producer	0	0
Mean days of intravenous therapy with study drug (SD)	5·6 (0.8)	6·1 (2.2)
Mean days of antibiotic therapy with study drugs (SD)	12·0 (4.8)	11·9 (1.9)
Oral therapy after intravenous therapy with study drug	29 (74.3)	29 (93.5)
Fosfomycin	29 (74.3)	0
Cefuroxime	0	28 (90.3)
Amoxicillin/clavulanic acid	0	1 (3.2)

SD: standard deviation. For variables definition, see Table 1 in the article.

[a] Data are expressed as No. (%) of participants unless otherwise indicated.

© 2022 Sojo-Dorado J et al. *JAMA Network Open.*

eTable 2. Baseline Characteristics of Patients in Modified Intention-to-Treat Population With Ceftriaxone-Resistant Isolates by Fosfomycin vs Meropenem[a]

Characteristic	Fosfomycin (n=39)	Meropenem (n=42)
Age in years, median (interquatile range)	72 (62-83)	74 (66-84)
Female gender	15 (38.5)	23 (54.8)
Charlson index, median (interquatile range)	2 (0-3)	2 (1-3)
Charlson index ≥3	15 (38.5)	16 (38.1)
Congestive heart failure	4 (10.3)	12 (28.6)
Chronic pulmonary Disease	8 (20.5)	7 (16.7)
Diabetes mellitus	10 (25.6)	11 (26.2)
Chronic renal Disease	7 (17.9)	12 (28.5)
Cancer	7 (17.9)	9 (21.4)
Full dependence for basic activities	1 (2.6)	6 (14.3)
Urinary catheter	13 (33.3)	13 (31.0)
Invasive procedure in the urinary tract in previous month	6 (15.4)	1 (2.4)
Immunosupressant drugs	4 (10.2)	8 (19.0)
Present infection		
Community-acquired infection	20 (51.3)	17 (40.5)
Healthcare-associated infection	10 (25.6)	17 (40.5)
Nosocomial infection	9 (23.1)	8 (19.0)
Low-urinary tract symptoms	22 (56.4)	24 (57.1)
Lumbar pain/tenderness	15 (38.5)	11 (26.2)
Severe sepsis at presentation	9 (23.1)	16 (38.1)
Pitt score, median (interquatile range)	1 (0-1.5)	1 (0-2)
Creatinine clearance ≤60 ml/min	14 (35.8)	13 (30.9)
Positive blood culture at recruitment day	6 (15.4)	5 (11.9)
Hydronephrosis in echography	6 (15.4)	2 (4.8)
Active treatment in ≤1 day since blood culture	22 (56.4)	25 (59.5)
Mean days until active treatment (SD)	0·5 (0.8)	0·6 (0.9)
Mean days until randomization (SD)	2·1 (0.4)	2·1 (0.4)
Removal/change of urinary catheter in ≤48h	7/13 (53.8)	13/13 (100)
Susceptibility of baseline *E. coli* (local laboratory)		
Amoxicillin	0	0
Amoxicillin-clavulanic acid	16 (41.0)	12 (28.6)
Piperacillin-tazobactam	26 (66.7)	28 (66.7)
Cefotaxime	0	0
Cefepime	3 (7.7)	2 (4.8)
Meropenem	29 (100)	42 (100)
Ciprofloxacin	7 (17.9)	6 (14.3)
Trimethoprim-sultamethoxazole	17 (43.6)	12 (28.6)
Amikacin	29 (74.4)	35 (83.3)
Fosfomycin	29 (100)	42 (100)
Baseline *E. coli* is ESBL producer	32/35 (91.4)	32/33 (96.9)
CTX-M-15 producer	18/32 (56.2)	20/32 (62.5)
Mean days of intravenous therapy with study drug (SD)	5·2 (0.9)	4·7 (0.8)
Mean days of antibiotic therapy with study drugs (SD)	10·8 (2.3)	11·9 (2.3)
Oral therapy after intravenous therapy with study drug	31 (79.4)	19 (45.3)
Fosfomycin	31 (79.4)	1 (2.3)[b]
Trimethoprim-sulfamethoxazole	0	7 (16.6)
Amoxicillin/clavulanic acid	0	6 (14.2)
Ciprofloxacin	0	5 (11.9)
Intramuscular ertapenem after intravenous therapy with study drug	0	13 (30.9)

SD: Standard deviation. For variables definition, see Table 1 in the article.
[a] Data are expressed as No. (%) of participants unless otherwise indicated. [b] Used by mistake.

© 2022 Sojo-Dorado J et al. *JAMA Network Open.*

eTable 3. Extended-Spectrum β-Lactamases Produced by Ceftriaxone-Resistant Isolates at Baseline

	Fosfomycin arm (n=39)	Meropenem arm (n=42)
Isolate not available	4	9
Non-ESBL-producer	3	1
CTX-M-15	18[a]	20
CTX-M-14	7	6
CTX-M-1	2	2
CTX-M-3	0	2
CTX-M-27	1	0
CTX-M-32	1	1
CTX-M-55	1	0
SHV-12	3[a]	1

[a] One isolate produced both CTX-M-15 and SHV-12

© 2022 Sojo-Dorado J et al. *JAMA Network Open.*

eTable 4. Patients Who Did Not Reach Clinical and Microbiological Cure at Test of Cure for Reasons Other Than Clinical or Microbiological Failure

**Patient**	**Arm**	**Age, gender**	**Underlying conditions**	**Reason for not reaching CMC at TOC**	**Comment**
1	Fosfomycin	68, male	Solid cancer	Urine culture not available	The patient had negative blood and urine culture by day5, and had criteria for clinical cure at TOC
2	Fosfomycin	82, female	Peripheral vascular disease	Withdrawn because of adverse event	The patient was withdrawn from the study 48 hours after first dose because of dyspnea, interpreted as heart failure and considered potentially related to the study drug; fosfomycin was stopped.
3	Fosfomycin	85, female	Chronic renal insufficiency	Withdrawn because of adverse event	The patient was withdrawn from the study 24 hours after first dose because of dyspnea, interpreted as heart failure and considered potentially related to the study drug; fosfomycin was stopped.
4	Fosfomycin	63, male	Diabetes mellitus	Urine culture not available	The patient had negative blood and urine by day 5 and had criteria for clinical cure at TOC
5	Fosfomycin	64, female	Chronic heart failure, chronic pulmonary disease	Urine culture not available	The patient had criteria for clinical cure at TOC
6	Fosfomycin	46, male	Urinary stones	TOC visit missing	Lost for follow-up after visit #4; the patient had negative urine and blood cultures by day 5 and was discharged after 6 days of IV therapy with oral fosfomycin.
7	Fosfomycin	63, female	Chronic heart failure, diabetes mellitus	TOC visit missing	Lost for follow-up after visit 2.
8	Fosfomycin	76, female	None	TOC visit missing	Lost for follow-up after visit 2.
9	Fosfomycin	83, male	Chronic heart failure, diabetes mellitus	Withdrawn because of adverse event	The patient was withdrawn from the study 48 hours after first dose because of dyspnea, interpreted as heart failure and considered potentially related to the study drug; fosfomycin was stopped.
10	Fosfomycin	86, male	Chronic pulmonary disease, chronic renal insufficiency	Withdrawn because of adverse events	The patient was withdrawn from the study 48 hours after first dose because of rash and mild dyspnea, interpreted as heart failure and considered potentially related to the study drug; fosfomycin was stopped.
11	Fosfomycin	38, female	None	Withdrawn because of adverse events	The patient was withdrawn from the study because of alithiasic cholecystitis 2 days after randomisation; antimicrobial treatment was changed.
12	Fosfomycin	58, male	Metastatic cancer	Withdrawn because of adverse event	The patient continued febrile at visit 3 and was withdrawn and changed to meropenem; the fever continued and was later considered to be related to cancer.
13	Comparator (meropenem)	37, female	None	TOC visit missing	Did not attend the TOC visit.
14	Comparator (meropenem)	54, female	Metastatic cancer, liver disease, chronic pulmonary disease	TOC visit missing	Lost for follow-up after visit #4; the patient had negative urine and blood cultures by day 5 and was discharged after 5 days of IV therapy with oral ciprofloxacin.

© 2022 Sojo-Dorado J et al. *JAMA Network Open.*

eTable 5. Analyses of Clinical or Microbiological Failure Rates at Test of Cure in Subgroups of Modified Intention-to-Treat Population

Subgroups	No./total no. (%)		Risk difference (1-sided 95% CI)[a]	1-sided P value
	Fosfomycin	Comparator		
Age ≤80 years	8/50 (16.0)	11/53 (20.7)	-4.7 (-∞ to 7.8)	0.26
Age >80 years	2/20 (10.0)	3/20 (15.0)	-5.0 (-∞ to 12.2)	0.31
Female	4/34 (11.8)	8/39 (20.5)	-8.7 (-∞ to 5.6)	0.15
Male	6/36 (16.7)	6/34 (17.6)	-0.9 (-∞ to 13.6)	0.45
Active empirical treatment	7/48 (14.6)	12/50 (24.0)	-9.4 (-∞ to 3.7)	0.11
Inactive empirical treatment	3/22 (13.6)	2/23 (8.6)	5.0 (-∞ to 20.3)	0.29
Charlson index ≤2[b]	8/48 (16.7)	9/51 (17.6)	-0.9 (-∞ to 11.7)	0.45
Charlson index >2[b]	2/22 (9.1)	5/22 (22.7)	-13.6 (-∞ to 4.5)	0.10
No severe sepsis[b]	9/55 (16.4)	9/51 (17.6)	-1.2 (-∞ to 11.2)	0.15
Severe sepsis[b]	1/15 (6.7)	5/22 (22.7)	-16.0 (-∞ to 4.3)	0.09
Community-acquired infection[b]	4/33 (12.1)	9/39 (23.0)	-10.9 (-∞ to 4.5)	0.11
Not community-acquired infection[b]	6/37 (16.2)	5/34 (14.7)	-1.5 (-∞ to 15.6)	0.43
Fosfomycin MIC ≤1 mg/L[c]	6/27 (22.4)	3/20 (15.0)	7.4 (-∞ to 26.5)	0.26
Fosfomycin MIC >1 mg/L[c]	3/33 (9.1)	8/37 (21.6)	-12.5 (-∞ to -1.8)	0.07

Abbreviations: MIC: minimum inhibitory concentration.
[a] The risk difference was calculated with a 1-sided 95% CI.
[b] For definitions, see Table 1.
[c] MIC was studied by agar microdilution in 117 available isolates.

© 2022 Sojo-Dorado J et al. *JAMA Network Open.*

eTable 6. Clinical and Microbiological Cure and Relapse Rates Among Patients Who Switched to Oral Drugs or Parenteral Ertapenem

**Oral/parenteral drug**	**Fosfomycin arm (n=60)**	**Comparators arm (n=61)**	**1-sided p value**
**Clinical and microbiological cure**			
All drugs	48/60 (80.0)	43/61 (77.0)	0.34
Fosfomycin trometamol	48/60 (80.0)	0/1 (0)[a]	-
Cefuroxime axetil	-	24/28 (85.7)	-
Amoxicillin-clavulanic acid	-	5/7 (71.4)	-
Trimethoprim-sulfamethoxazole	-	5/7 (71.4)	-
Ciprofloxacin	-	4/5 (80.0)	-
Ertapenem (parenteral)	.	9/13 (61.5)	-
**Recurrence**			
All drugs	8/60 (13.3)	4 /61 (8.1)	0.17
Fosfomycin trometamol	8/60 (13.3)	1/1 (100)[a]	-
Cefuroxime axetil	-	0/28 (0)	-
Amoxicillin-clavulanic acid	-	1/7 (14.2)	-
Trimethoprim-sulfamethoxazole	-	1/7 (14.2)	-
Ciprofloxacin	-	0/4 (0)	-
Ertapenem (parenteral)	-	2/13 (15.3)	-

[a] Prescribed by mistake

© 2022 Sojo-Dorado J et al. *JAMA Network Open.*

eTable 7. Multivariate Analysis of Variables Associated With Clinical and Microbiological Cure in Modified Intention-to-Treat Population

Variable	Adjusted OR (95% CI)	P value
Fosfomycin arm	0.55 (0.24-1.21)	0.14
Ceftriaxone-resistant isolate	0.32 (0.11-0.87)	0.02
Days until first drug administered	1.38 (0.96-1.99)	0.07
Vesical catheter	2.78 (1.05-7.39)	0.04

© 2022 Sojo-Dorado J et al. *JAMA Network Open.*

eTable 8. Adverse Events Reported

Data are no. of patients (percentage) for the two first columns, and no. of patients for other columns.

	**Fosfomycin (n=70)**	**Comparators (n=73)**	**Fosfomycin arm, cephalosporin-susceptible isolates (n=31)**	**Fosfomycin arm, cephalosporin-resistant isolates (n=39)**	**Ceftriaxone arm (n=31)**	**Meropenem arm (n=42)**
**Gastrointestinal disorders**						
Nausea/vomiting	4 (5.7)	1 (1.3)	3	1	0	1
Dyspepsia	1 (1.4)	0	1	0	0	0
Diarrhea	7 (10.0)	8 (10.9)	2	5	4	4
Pancreatitis	0	1 (1.3)	0	0	0	1
Abdominal pain	3 (4.2)	0	1	2	0	0
Dry mouth	1 (1.4)	0	0	1	0	0
Oral mucositis	1 (1.4)	0	0	1	0	0
Constipation	2 (2.8)	0	1	1	0	0
**Infections and infestations**						
Respiratory tract	2 (2.8)	5 (6.8)	0	2	1	4
Skin soft tissue	1 (1.4)	1 (1.3)	0	1	0	1
Primary bacteremia/sepsis with unknown source	1 (1.4)	2 (2.7)	1	0	0	2
Mucosal infection	2 (2.8)	3 (4.1)	1	1	3	0
Biliary tract	1 (1.4)	0	0	1	0	0
Clostridioides difficile infection	1 (1.4)	0	0	1	0	0
**General disorders and administration site conditions**						
Fatigue	1 (1.4)	2 (2.7)	1	0	1	1
Edema	10 (14.2)	10 (13.6)	5	5	4	6
Phlebitis or extravasation (venous catheter site)	10 (14.2)	4 (5.4)	6	4	2	2
Fever, non-infection related	1 (1.4)	0	0	1	0	0

© 2022 Sojo-Dorado J et al. *JAMA Network Open.*

Death, unknown cause	0	1 (1.3)	0	0	0	1
**Blood and lymphatic system disorders**						
Bleeding	2 (2.8)	5 (6.8)	1	1	0	5
Anemia	3 (4.2)	1 (1.3)	1	2	0	1
Thrombocytopenia	0	1 (1.3)	0	0	1	0
Thrombocytosis	1 (1.4)	0	1	0	0	0
**Nervous system disorders**						
Syncope/presyncope	1 (1.4)	1 (1.3)	0	1	0	1
Dizziness, vertigo	0	3 (4.1)	0	0	2	1
Paresthesia	0	1 (1.3)	0	0	1	0
Headache	2 (2.8)	1 (1.3)	1	1	0	1
Disgeusia	1 (1.4)	0	0	1	0	0
**Respiratory, thoracic and mediastinal disorders**						
Aspiration	0	1 (1.3)	0	0	0	1
Pharynx pain	1 (1.4)	1 (1.3)	1	0	1	0
**Renal and urinary disorders**						
Renal insufficiency	2 (2.8)	2 (2.7)	0	2	1	1
Urinary tract obstruction	2 (2.8)	3 (4.1)	2	0	1	2
Abacterial prostatitis	1 (1.4)	0	1	0	0	0
Urethral discomfort, non-UTI related	4 (5.7)	1 (1.3)	2	2	0	1
**Musculoeskeletal and connective tissue disorders**						
Limb/back pain	6 (8.5)	2 (2.7)	2	4	2	0
Bone fracture	0	2 (2.7)	0	0	0	2
**Cardiac disorders**						
Heart failure	6 (8.5)	2 (2.7)	2	4	1	1
Heart murmur	1 (1.4)	0	1	0	0	0
**Investigations**						
AST, ALT elevation	4 (5.7)	6 (8.2)	3	1	3	3
Hypernatremia	1 (1.4)	0	0	1	0	0
Hypokalemia	8 (11.4)	0	3	5	0	0

© 2022 Sojo-Dorado J et al. *JAMA Network Open.*

Hyperkalemia	1 (1.4)	0	0	1	0	0
Hypomagnesemia	1 (1.4)	0	1	0	0	0
Hypoalbuminemia	2 (2.8)	0	0	2	0	0
**Injury, poisoning and procedural complications**						
Fall	0	2 (2.7)	0	0	0	2
Traumatism in urethral catheter	1 (1·4)	0	1	0	0	0
**Skin and subcutaneous tissue disorders**						
Rash/urticarial	3 (4.2)	1 (1.3)	1	2	0	1
Pruritus	1 (1.4)	1 (1.3)	1	0	1	0
Ulcer	2 (2.8)	1 (1.3)	0	2	1	0
Cutaneous herpes	0	1 (1.3)	0	0	0	1
Dermatitis, nephrostomy insertion site	1 (1.4)	0	1	0	0	0
**Endocrine disorders**						
Hyperglucemia	1 (1.4)	0	0	1	0	0
**Vascular disorders**						
Hypotension	1 (1.4)	0	1	0	0	0
Hypertension	2 (2.8)	0	2	0	0	0
**Neoplasms benign, malignant and unspecified**						
Cancer progression	2 (2.8)	1 (1.3)	1	1	0	1
**Psychiatric disorders**						
Anxiety	2 (2.8)	1 (1.3)	1	1	0	0

© 2022 Sojo-Dorado J et al. *JAMA Network Open.*

eTable 9. Description of Serious Adverse Events

Defined as prolonging or needing hospitalization, is life-threatening, cause death or cause permanent or significant disability.

Patient	Arm (date of randomisation)	Age, gender	Underlying conditions	Adverse event (start date)	Outcome	Relation to study drug
1	Meropenem (29/7/14)	78, female	Chronic heart failure, chronic renal disease	Vertebral fracture after fall secondary to pre-syncope (2/9/14)	Recovered with sequela	Unrelated
2	Fosfomycin (13/3/15)	87, female	Chronic renal disease, diabetes mellitus	Biliary tract infection (27/4/15)	Recovered	Unrelated
3	Fosfomycin (16/4/15)	85, female	Chronic renal insufficiency	Heart failure (17/4/15)	Recovered	Probably related
4	Fosfomycin (8/5/15)	89, male	Chronic pulmonary disease, diabetes mellitus, ischemic heart disease	Urticaria (11/5/15)	Recovered	Probably related
5	Fosfomycin (27/5/15)	81, male	Chronic heart failure, ischemic heart disease, chronic pulmonary disease, metastatic cancer	Heart failure (29/5/15)	Recovered. Fosfomycin was stopped	Probably related
				Hypercalcemia, cancer-related (08/6/15)	Death	Unrelated
6	Meropenem (16/6/15)	80, male	Solid cancer	Pressure ulcer infection (7/7/15)	Recovered with sequela	Unrelated
7	Meropenem (1/7/15)	86, female	Dementia, diabetes mellitus	Acute renal insufficiency (13/8/15)	Fosfomycin was stopped. Recovered	Unrelated
8	Meropenem (28/10/2015	48, female	Cancer	Sepsis, source unclear (11/10/2015	Recovered	Unrelated
9	Fosfomycin (18/2/16)	71, male	Diabetes mellitus, chronic renal insufficiency, chronic pulmonary disease	Bronchitis with dyspnea (27/4/16)	Recovered	Unrelated
10	Fosfomycin (3/6/16)	81, male	Chronic heart failure, chronic pulmonary disease, chronic renal insufficiency	Hypokaliemia (7/6/16)	Recovered	Probably related
11	Meropenem (30/3/2016)	48, male	Chronic renal insufficiency	Anemia (16/4/16)	Recovered	Unrelated
12	Fosfomycin (14/6/16)	86, male	Chronic pulmonary disease, chronic renal insufficiency	Heart failure and rash (16/6/16)	Fosfomycin was stopped. Recovered	Probably related
13	Fosfomycin (11/8/16)	68, male	Metastasic cancer, diabetes mellitus, chronic renal disease	Progression of cancer (4/10/16)	Death	Unrelated
14	Meropenem (19/9/16)	89, male	Leukemia, chronic heart failure, chronic pulmonary disease, dementia	Death, unknown cause (2/11/2020)	Death	Unrelated
15	Fosfomycin (29/9/16)	83, male	Chronic heart failure, diabetes mellitus	Heart failure (3/10/16)	Fosfomycin was stopped. Recovered with sequela	Probably related
				Dyspnea, attributed to heart failure (18/10/16)	Death	Unrelated
16	Fosfomycin (28/12/16)	58, male	Metastatic cancer	Persistent fever, cancer-related (28/12/16)	Fosfomycin was stopped. Recovered	Unrelated
17	Meropenem (2/2/17)	90, female	Chronic heart failure, chronic renal insufficiency	Diarrea (8/2/17))	Recovered	Probably related
				Heart failure (28/2/2020)	Recovered	Unrelated
18	Meropenem (2/2/17)	79, female	Dementia chronic pulmonary disease	Hip fracture (26/2/17)	Recovered	Unrelated

© 2022 Sojo-Dorado J et al. *JAMA Network Open.*

19	Fosfomycin (24/2/17)	58, male	Diabetes mellitus, cancer	Fever after nephrostomy manipulation, urine culture with mixed flora (11/3/17)	Recovered	Unrelated
20	Fosfomycin (3/3/17)	84, female	None	Bilateral psoas hematoma (12/4/17)	Recovered	Unrelated
21	Meropenem (6/6/17)	73, male	Leukemia	Respiratory tract infection (30/6/17)	Death	Unrelated
22	Meropenem (18/9/17)	75, female	Dementia, diabetes mellitus, chronic heart failure	Acute pancreatitis (11/10/17)	Recovered	Unrelated
				Hydronephrosis secondary to litiasis not present at admission (5/10/17)	Recovered	Unrelated
23	Fosfomycin (20/6/18)	82, female	Peripheral vascular disease	Heart failure (22/6/18)	Fosfomycin was stopped. Recovered	Probably related

© 2022 Sojo-Dorado J et al. *JAMA Network Open.*

eTable 10. Ceftriaxone-Resistant Bacteria Isolated After Treatment

Isolates	All patients		Ceftriaxone-susceptible *E. coli* at baseline		Ceftriaxone-resistant *E. coli* at baseline	
	Fosfomycin (n=70)	Comparators (n=73)	Fosfomycin (n=31)	Ceftriaxone (n=31)	Fosfomycin (n=39)	Meropenem (n=42)
**ALL CEFTRIAXONE-RESISTANT BACTERIA**	20 (29·5)	27 (36.9)	4 (12.9)	6 (19.3)	16 (41.0)	21 (50.0)
**Ceftriaxone-resistant Enterobacterales**	14 (20.0)	16 (21.9)	0 (0)	2 (6.4)	14 (35.8)	14 (33.3)
Escherichia coli	14	15	0	2	14	13
Other Enterobacterales	0	1	0	0	0	1
**Non-fermentative gram negative bacteria**	3 (4.2)	4 (5.4)	2 (6.4)	1 (3.2)	1 (2.5)	3 (7.1)
Pseudomonas aeruginosa	2	2	1	1	1	1
Acinetobacter baumannii	0	1	0	0	0	1
Stenotrophomonas maltophilia	1	1	1	0	0	1
***Enterococcus* spp.**	3 (4.2)	7 (9.5)	2 (6.4)	3 (9.6)	1 (2.5)	4 (9.5)
**ALL MEROPENEM-RESISTANT BACTERIA**	2 (2.8)	3 (4.1)	1 (3.2)	0	1 (2.5)	3 (7.1)
**Non-fermentative gram negative bacteria**	1 (1.4)	2 (2.7)	1 (3.2)	0	0	2 (4.7)
Acinetobacter baumannii	0	1	0	0	0	1
Stenotrophomonas maltophilia	1	1	1	0	0	1
***Enterococcus* spp.**	1 (1.4)	1 (1.3)	0	0	1 (2.5)	1 (2.3)
**ALL FOSFOMYCIN-RESISTANT BACTERIA**	8 (11.4)	6 (8.2)	2 (6.4)	1 (3.2)	6 (6.6)	5 (11.9)
**Fosfomycin-resistant Enterobacterales**	7 (10.0)	3 (4.1)	1 (3.2)	1 (3.2)	6 (6.6)	2 (8.3)
Escherichia coli	2	2	0	0	2	2
Other Enterobacterales	5	1	1	1	4	0
**Non-fermentative gram negative bacteria**	1 (1.4)	3 (4.1)	1 (3.2)	0	0	3 (7.1)
Pseudomonas aeruginosa	0	1	0	0	0	1
Acinetobacter baumannii	0	1	0	0	0	1
Stenotrophomonas maltophilia	1	1	1	0	0	1

Data are number of patients (percentage); percentages are specified only for main bacterial groups.

© 2022 Sojo-Dorado J et al. *JAMA Network Open.*
