# Supplementary material for: Effectiveness of Fosfomycin for the Treatment of Multidrug-Resistant Escherichia coli Bacteremic Urinary Tract Infections: A Randomized Clinical Trial
Source: JAMA Netw Open. 2022 Jan 13;5(1):e2137277. doi: 10.1001/jamanetworkopen.2021.37277 (PMC8759008; doi:10.1001/jamanetworkopen.2021.37277)
Supplement: Supplement 3. — Nonauthor Collaborators [file jamanetwopen-e2137277-s003.pdf]

Supplemental Online Content: Nonauthor Collaborators

*Indicates required information. Only first name, last name, and suffix will appear in PubMed.

***Group Name(s): REIPI-GEIRAS-FOREST group**							
***First Name and Middle Initial(s)**	***Last Name**	***Suffix (eg, Jr, III)**	Academic Degrees	Institution	Location (city, state/province, country)	Role or Contribution, eg, chair, principal investigator	Group (if more than 1 Group listed in the byline) and/or Subgroup (eg, Steering Committee)
Benito	Almirante			Hospital Vall d'Hebrón	Barcelona	Member of the data safety monitoring board	
Mario	Fernández			Hospital 12 de Octubre	Madrid	Member of the data safety monitoring board	
José Ramón	Paño-Pardo			Hospital Lozano Blesa	Zaragoza	Member of the data safety monitoring board	
Marina	De Cueto			Hospital Universitario Virgen Macarena	Sevilla	Patients recruitment, data collection, review of draft	
Pilar	Retamar-Gentil			Hospital Universitario Virgen Macarena	Sevilla	Patients recruitment, data collection, review of draft	
Luis Eduardo	López-Cortés			Hospital Universitario Virgen Macarena	Sevilla	Patients recruitment, data collection, review of draft	
Belén	Gutiérrez-Gutiérrez			Hospital Universitario Virgen Macarena	Sevilla	Patients recruitment, data collection, review of draft	
Fernando	Docobo			Hospital Universitario Virgen Macarena	Sevilla	Microbiological studies	
Irene	Borreguero			Hospital Universitario Virgen Macarena	Sevilla	Monitoring coordination	
Manuel	Camean			Hospital Universitario Virgen Macarena	Sevilla	Pharmacy coordination	

Supplemental Online Content: Nonauthor Collaborators

*Indicates required information. Only first name, last name, and suffix will appear in PubMed.

***First Name and Middle Initial(s)**	***Last Name**	***Suffix (eg, Jr, III)**	Academic Degrees	Institution	Location (city, state/province, country)	Role or Contribution, eg, chair, principal investigator	Group (if more than 1 Group listed in the byline) and/or Subgroup (eg, Steering Committee)
Encarnación	Moral-Escudero			Hospital Clínico Universitario Virgen de la Arrixaca	Murcia	Patients recruitment, data collection, review of draft	
Ana	Pareja-Rodríguez de Vera			Hospital Clínico Universitario Virgen de la Arrixaca	Murcia	Patients recruitment, data collection, review of draft	
María del Carmen	Martínez-Toldos			Hospital Clínico Universitario Virgen de la Arrixaca	Murcia	Patients recruitment, data collection, review of draft	
Ana	Blázquez-Abellán			Hospital General Universitario Santa Lucía	Cartagena, Murcia	Patients recruitment, data collection, review of draft	
Alba	Belles-Belles			Hospital Universitari Arnau de Vilanova	Lleida	Patients recruitment, data collection, review of draft	
María Fernanda	Ramírez-Hidalgo			Hospital Universitari Arnau de Vilanova	Lleida	Patients recruitment, data collection, review of draft	
Beatriz	Mirelis			Hospital de la Santa Creu i Sant Pau	Barcelona	Patients recruitment, data collection, review of draft	
Esther	Calbo			Hospital Universitari Mútua Terrassa	Terrassa, Barcelona	Patients recruitment, data collection, review of draft	

Supplemental Online Content: Nonauthor Collaborators

*Indicates required information. Only first name, last name, and suffix will appear in PubMed.

***First Name and Middle Initial(s)**	***Last Name**	***Suffix (eg, Jr, III)**	Academic Degrees	Institution	Location (city, state/province, country)	Role or Contribution, eg, chair, principal investigator	Group (if more than 1 Group listed in the byline) and/or Subgroup (eg, Steering Committee)
Mariona	Xercavins			CatLab	Viladecavalls, Barcelona	Patients recruitment, data collection, review of draft	
Irene	Gracia-Ahufinger			Hospital Universitario Reina Sofía	Córdoba	Patients recruitment, data collection, review of draft	
Angela M.	Cano-Yuste			Hospital Universitario Reina Sofía	Córdoba	Patients recruitment, data collection, review of draft	
Laura	Guío			Hospital Universitario Cruces, Instituto de Investigación Biocruces	Baracaldo, Vizcaya	Patients recruitment, data collection, review of draft	
Jose Luis	Hernandez			Hospital Universitario Cruces, Instituto de Investigación Biocruces	Baracaldo, Vizcaya	Patients recruitment, data collection, review of draft	
Carlos	Pigrau-Serrallach			Hospital Universitario Vall d' Hebron	Barcelona	Patients recruitment, data collection, review of draft	
Belen	Viñado-Pérez			Hospital Universitario Vall d' Hebron	Barcelona	Patients recruitment, data collection, review of draft	

Supplemental Online Content: Nonauthor Collaborators

*Indicates required information. Only first name, last name, and suffix will appear in PubMed.

***First Name and Middle Initial(s)**	***Last Name**	***Suffix (eg, Jr, III)**	Academic Degrees	Institution	Location (city, state/province, country)	Role or Contribution, eg, chair, principal investigator	Group (if more than 1 Group listed in the byline) and/or Subgroup (eg, Steering Committee)
Mireia	Puig Asensio			Hospital Universitario Vall d' Hebron	Barcelona	Patients recruitment, data collection, review of draft	
Carmen	Ardanuy			Hospital Universitario de Bellvitge	Barcelona	Patients recruitment, data collection, review of draft	
Miquel	Pujol			Hospital Universitario de Bellvitge	Barcelona	Patients recruitment, data collection, review of draft	
Dácil	García-Rosado			Hospital Universitario de Canarias	La Laguna	Patients recruitment, data collection, review of draft	
Concepción	Gil-Anguita			Hospital Marina Baixa	Villajoyosa, Alicante	Patients recruitment, data collection, review of draft	
Ana	Siverio			Laboratori de Referéncia a Catalunya	Barcelona	Patients recruitment, data collection, review of draft	
Adelina	Gimeno-Gascón			Hospital General Universitario de Alicante	Alicante	Patients recruitment, data collection, review of draft	

Supplemental Online Content: Nonauthor Collaborators

*Indicates required information. Only first name, last name, and suffix will appear in PubMed.

***First Name and Middle Initial(s)**	***Last Name**	***Suffix (eg, Jr, III)**	Academic Degrees	Institution	Location (city, state/province, country)	Role or Contribution, eg, chair, principal investigator	Group (if more than 1 Group listed in the byline) and/or Subgroup (eg, Steering Committee)
Vicente	Boix-Martínez			Hospital General Universitario de Alicante	Alicante	Patients recruitment, data collection, review of draft	
Sergio	Reus-Bañuls			Hospital General Universitario de Alicante	Alicante	Patients recruitment, data collection, review of draft	
Iván	Agea-Durán			Hospital General Universitario de Alicante	Alicante	Patients recruitment, data collection, review of draft	
Carmen	Fariñas			Hospital Universitario Marqués de Valdecilla	Santander	Patients recruitment, data collection, review of draft	
Begoña	Palop			Hospital Regional Universitario de Málaga	Málaga	Patients recruitment, data collection, review of draft	
Helem	Vilchez			Hospital Son Espases	Palma de Mallorca	Patients recruitment, data collection, review of draft	
José Antonio	Lepe			Hospital Universitario Virgen del Rocío	Sevilla	Patients recruitment, data collection, review of draft	

Supplemental Online Content: Nonauthor Collaborators

*Indicates required information. Only first name, last name, and suffix will appear in PubMed.

***First Name and Middle Initial(s)**	***Last Name**	***Suffix (eg, Jr, III)**	Academic Degrees	Institution	Location (city, state/province, country)	Role or Contribution, eg, chair, principal investigator	Group (if more than 1 Group listed in the byline) and/or Subgroup (eg, Steering Committee)
María Victoria	Gil-Navarro			Hospital Universitario Virgen del Rocío	Sevilla	Patients recruitment, data collection, review of draft	
Rafael	San-Juan			Hospital Universitario Doce de Octubre	Madrid	Patients recruitment, data collection, review of draft	
Fernando	Chaves			Hospital Universitario Doce de Octubre	Madrid	Patients recruitment, data collection, review of draft	
Rosa	Escudero			Hospital Universitario Ramón y Cajal	Madrid	Patients recruitment, data collection, review of draft	
Francesca	Gioia			Hospital Universitario Ramón y Cajal	Madrid	Patients recruitment, data collection, review of draft	
Ana María	Sánchez-Díaz			Hospital Universitario Ramón y Cajal	Madrid	Patients recruitment, data collection, review of draft	
Ana	Cañas-Pedrosa			Hospital Universitario de Gran Canarias Dr. Negrín	Las Palmas de Gran Canaria	Patients recruitment, data collection, review of draft	

Supplemental Online Content: Nonauthor Collaborators

*Indicates required information. Only first name, last name, and suffix will appear in PubMed.

***First Name and Middle Initial(s)**	***Last Name**	***Suffix (eg, Jr, III)**	Academic Degrees	Institution	Location (city, state/province, country)	Role or Contribution, eg, chair, principal investigator	Group (if more than 1 Group listed in the byline) and/or Subgroup (eg, Steering Committee)
Nayra	Sangil-Monroy			Hospital Universitario de Gran Canarias Dr. Negrín	Las Palmas de Gran Canaria	Patients recruitment, data collection, review of draft	
Carla	Toyas-Miazza			Hospital Royo Villanova	Zaragoza	Patients recruitment, data collection, review of draft	
