# Supplementary material for: Effectiveness of Fosfomycin for the Treatment of Multidrug-Resistant Escherichia coli Bacteremic Urinary Tract Infections: A Randomized Clinical Trial
Source: JAMA Netw Open. 2022 Jan 13;5(1):e2137277. doi: 10.1001/jamanetworkopen.2021.37277 (PMC8759008; doi:10.1001/jamanetworkopen.2021.37277)
Supplement: Supplement 4. — Data Sharing Statement [file jamanetwopen-e2137277-s004.pdf]

Data Sharing Statement

Sojo-Dorado J, López-Hernández I, Rosso-Fernandez C, et al; REIPI-GEIRAS-FOREST group. Effectiveness of fosfomycin for the treatment of multidrug-resistant Escherichia coli bacteremic urinary tract infections: a randomized clinical trial. *JAMA Netw Open*. 2022;5(1):e2137277. doi:10.1001/jamanetworkopen.2021.37277

Data
Data available: Yes
Data types: Deidentified participant data, Data dictionary
How to access data: Acces via email to jesusrb@us.es
When available: With publication

Supporting Documents
Document types: Statistical/analytic code, Informed consent form
How to access documents: Via email to jesusrb@us.es
When available: With publication

Additional Information
Who can access the data: Researchers whose proposed used of the data has been approved **Types of analyses:** Meta-analysis, seconday analyses
Mechanisms of data availability: Via email to jesusrb@us.es
Any additional restrictions: Previous agreement signed with correspondoing author's institution

Individual, anonymized data would be shared after a signed agreement with Fundación Pública Andaluza para la Gestión de la Investigación en Salud de Sevilla if requested with the objective of performing a meta-analysis with individual patients' data. Requests should be submitted to the corresponding author. Interested researchers should obtain the approval of the Ethic Committee at Hospital Universitario Virgen Macarena and Virgen del Rocío. A database in SPSS file with the requested data and a dictionary of terms would be provided.

© 2022 Sojo-Dorado J et al. JAMA Network Open.
